# Measuring the quality of patient-provider relationships in serious illness: A scoping review

**DOI:** 10.1177/02692163251315304

**Published:** 2025-02-06

**Authors:** Karen Wassef, Kristine Ma, Brigitte N Durieux, Tyler L Brown, Joanna Paladino, Sally Thorne, Justin J Sanders

**Affiliations:** 1 Department of Family Medicine, Faculty of Medicine and Health Sciences, McGill University, Montréal, QC, Canada; 2 Goldman Herzl Family Practice Centre, Jewish General Hospital, Montréal, QC, Canada; 3 Department of Psychosocial Oncology and Palliative Care, Dana-Farber Cancer Institute, Boston, MA, USA; 4 Department of Oncology, Faculty of Medicine and Health Sciences, McGill University, Montréal, QC, Canada; 5 Center for Aging and Serious Illness, Mongan Institute, Massachusetts General Hospital, Boston, MA, USA; 6 School of Nursing, University of British Columbia, Vancouver, BC, Canada; 7 Research Institute, McGill University Health Centre, Montréal, QC, Canada

**Keywords:** Healthcare quality, palliative care, professional-patient relations, review

## Abstract

**Background:**

People affected by serious illness face several threats to their well-being: physical symptoms, psychological distress, disrupted social relations, and spiritual/existential crises. Relationships with clinicians provide a form of structured support that promotes shared decision-making and adaptive stress coping. Measuring relationship quality may improve quality assessment and patient care outcomes. However, researchers and those promoting quality improvement lack clear guidance on measuring this.

**Aim:**

To identify and assess items from valid measures of patient-provider relationship quality in serious illness settings for guiding quality assessment.

**Design:**

Scoping review.

**Data sources:**

We identified peer-reviewed, English-language articles published from 1990 to 2023 in CINAHL, Embase, and PubMed. Eligible articles described the validation of measures assessing healthcare experiences of patient populations characterized by serious illness. We used Clarke et al.’s theory of relationship quality to assess relationship-focused items.

**Results:**

From 3868 screened articles, we identified 101 publications describing 47 valid measures used in serious illness settings. Measures assessed patients and other caregivers. We determined that 597 of 2238 items (26.7%) related to relationships. Most measures (*n* = 46) included items related to engaging the patient as a whole person. Measures evaluated how providers promote information exchange (*n* = 35), foster therapeutic alliance (*n* = 35), recognize and respond to emotion (*n* = 27), and include patients in care-related decisions (*n* = 23). Few instruments (*n* = 9) assessed patient self-management and navigation.

**Conclusions:**

Measures include items that assess patient-provider relationship quality in serious illness settings. Researchers may consider these for evaluating and improving relationship quality, a patient-centered care and research outcome.

**What is already known about the topic?**

- Patient-provider relationships can influence patients’ health outcomes and their satisfaction with care.
- Multiple factors may contribute to the quality of patient-provider relationships, including mutual trust, empathy, and effective communication.
- There has been no systematically informed review on how to measure relationship quality in serious illness settings.

**What this paper adds?**

- This review systematically identifies and presents relationship-focused items from previously validated measurement tools used in settings of serious illness to assess aspects that are pertinent to the patient-provider relationship.
- We categorized these items using six empirically derived domains related to the quality of patient-provider relationships.

**Implications for practice, theory, or policy**

- Relationship quality may be considered a patient-centered outcome for research or a mediator of high-quality care.
- Identified measures may have utility in quality improvement initiatives and can inform clinical training programs aimed at improving patient-provider relationships.
- Further research is needed to explore the role of culture and the dynamic nature of relationships in assessments of relationship quality.

## Introduction

Patients affected by serious illness experience multiple forms of suffering across physical, psychosocial, and spiritual dimensions.^1–5^ Cassell’s^6^ formative definition depicted suffering as severe distress that occurs when a perceived threat risks compromising one’s sense of dignity and personal wholeness. Research suggests that relationships with others restore dignity and wholeness, thereby ameliorating suffering.^2,6–8^ The quality of relationships may mediate the impact of medical interventions on patient experiences.^6^ Therefore, understanding the quality of relationships and how to measure them presents a critical opportunity to improve healthcare quality for people affected by serious illness.

Within palliative care, clinicians explicitly focus on building relationships to aid shared decision-making and facilitate adaptive stress coping.^8–12^ Palliative care adopts a holistic, patient-centered approach to suffering and explicitly focuses on healing relationships.^13,14^ These relationships hold the potential to relieve suffering and encourage patients to adaptively cope with their illness from early diagnosis to end-of-life.^15^ The relational focus of palliative care accords with social cognitive neuroscience literature suggesting significant benefits of relationships.^8,16,17^ Facilitating patient-provider connectedness activates brain regions associated with effective stress and emotional regulation,^17^ which may contribute to a greater sense of patient well-being.^15^

Despite the potential importance of these relationships, our limited knowledge of measuring relationship quality may undermine our efforts to use this outcome as an indicator of quality improvement. With the goal of enhancing quality assessment in serious illness settings, we conducted a scoping review of valid measures that evaluate the quality of patient-provider relationships.

## Methods

We conducted a scoping review of the literature on valid measures of patient-provider relationship quality within settings of serious illness. We used a five-stage approach articulated by Arksey and O’Malley^18^: (1) identify the research question, (2) identify relevant studies, (3) select studies, (4) chart the data, and (5) collate, summarize, and report the results. We also followed the Preferred Reporting Items for Systematic Reviews and Meta-Analyses Extension for Scoping Reviews (PRISMA-ScR).^19^

### Identifying the research question

This review aims to identify peer-reviewed validation studies of tools that measure relationship quality between healthcare providers and patients with serious illness. As such, the research question guiding this review is: “*What questions or survey items from valid measures can assess the quality of patient-provider relationships in the context of serious illness?*”

### Eligibility criteria

We included studies in our review that quantitatively validated measures of healthcare experience, with specific attention to relationships between seriously ill patients and their healthcare providers. Table 1 displays the inclusion and exclusion criteria.

**Table 1.** Initial inclusion and exclusion criteria.^a^

Criterion	Inclusion	Exclusion
Period	1 January 1990–1 May 2023	Published before 1990
Language	English	Language other than English
Type of studies	Quantitative and mixed method studies published in peer-reviewed journals	Qualitative studies, letters, comments, conference abstracts, editorials, theses, gray literature (i.e. any publication that has not been peer-reviewed)
Type of participants	Patients with serious illness and/or their surrogates, caregivers, or relatives who have evaluated the patient-provider relationship Healthcare providers involved in a setting of serious illness	Patients who do not have a serious illness Healthcare providers or other personnel who do not support patients in serious illness settings Patients below 18 years old Pediatric clinical settings
Type of outcomes	Quantitative psychometric validation of instruments (e.g. surveys, questionnaires) measuring patient-provider relationship quality in settings of serious illness	Studies that are unrelated to quantitative validation of measures of patient-provider relationship quality in settings of serious illness

a We applied additional inclusion/exclusion criteria when selecting measure items during data extraction (see *Synthesis of Results Section*).

### Information sources

We searched CINAHL, Embase, and PubMed, which include relevant journals in health and medicine, palliative care, allied health, and instrument validation. We manually searched reference lists of included articles to identify potentially relevant studies that were not retrieved by the search strategy (i.e. snowball sampling).

### Search strategy

In coordination with a liaison librarian, we developed and piloted in PubMed an initial search strategy using relevant Medical Subject Headings (MeSH) and keywords. Our search concepts include: serious illness, patient-provider relationships, measurement tools, and validation. We defined “serious illness” as a health condition that carries a high mortality risk and negatively impacts quality of life.^20^ We included a range of healthcare providers in our search strategy given that serious illness care, including palliative care, adopts a multidisciplinary approach.^21^ We maximized the sensitivity and specificity of our search to obtain a comprehensive range of relevant studies. We used the Boolean operator “or” to broaden the search by combining synonyms pertaining to each concept and added a “validation” concept to make the search more specific.^22^ We excluded studies that validate measures related to providers’ skills and aptitude as we aimed to focus on patient experiences of these relationships. We limited our search to articles published in the English language within the last 30 years because of increased attention to populations with serious illness since that time.

We applied a refined and final search strategy in CINAHL, Embase, and PubMed on May 1, 2023 (Supplemental Material 1). Articles obtained from each database were uploaded to EndNote (Version 20.4).^23^ These were exported and uploaded to Covidence.^24^ We manually removed duplicates that Covidence did not identify during the study selection stage.

### Study selection

Two authors (KW and KM) completed the review of titles and abstracts to ensure inclusion of all relevant studies.^25^ We verified that the eligibility criteria can be consistently applied by performing a calibration exercise prior to independent screening. We first discussed a sample of test articles to discern the types of articles to include. These discussions generated a list of articles to be included or excluded which we compared other articles to during the first phase of title and abstract screening. We individually evaluated titles and abstracts of 3868 studies to identify those which were relevant to the research question and which met eligibility criteria. We convened and resolved by consensus articles involving an initial disagreement and reviewed these with another author (JS) when uncertain.

After completing title and abstract screening, two authors (KW and KM) reviewed the full texts of a random sample of 11 included studies to ensure standardized application of the eligibility criteria. After reaching a minimum threshold of 80% agreement on these, we independently reviewed the full texts of all 109 studies in our Covidence record, from which we extracted 63 studies as relevant. After searching the reference lists of these, we identified and included 38 eligible studies that were not retrieved by the search strategy. We excluded studies if we could not access the full content of the measures and could not reach the respective authors.

### Data charting process

We developed a data charting form on Microsoft Word for extracting relevant variables that characterize the range of evidence obtained. These variables include the study location, type of respondent and healthcare provider, clinical setting, and relationship-focused items from the valid measures. KW then independently charted the data, discussed the results with JS to verify the information extracted, and continuously updated the data charting form in an iterative process based on these discussions. Supplemental Material 2 presents the data extraction tables according to the type of respondent.

### Synthesis of results

To promote rigor and consistency, we adopted an empirically informed framework to guide our conceptualization of measures assessing relationship quality in settings of serious illness. We used Clarke et al.’s^26^ six defining elements of therapeutic relationships between patients and health professionals (Table 2) to synthesize the evidence we found. These elements were used for two primary reasons: (1) they were informed by literature on patient-centered care and communication, which are essential pillars of care approaches to serious illness, and (2) they were derived to guide development of quality assessment tools which reflects our focus on measures of relationship quality. We presented the distribution of items we included across these six elements in Supplemental Material 3.

**Table 2.** Elements and sub-elements of relationship quality.^26^

Elements	Sub-elements		
(1) Engaging the patient as a whole person (2) Recognizing and responding to emotions (3) Fostering therapeutic alliance (4) Promoting information exchange (5) Sharing decision-making (6) Enabling self-management and patient navigation	Adopting a biopsychosocial perspective Identifying and understanding emotional cues Establishing and sustaining trust Facilitating information exchange Finding common ground Enabling patient self-management	Respecting the individual, their needs and preferences Validating and reacting to emotional cues Sharing power with the patient Ensuring information retention Engaging patients in their care Enhancing patient navigation	Acknowledging the relational patient

We centered our review on more explicit relational aspects represented by the measures. We looked to identify measures that assessed patient, surrogate, or caregiver experiences about providers’ personal attributes and actions as well as other outcomes that seemed likely sensitive to the quality of the patient-provider relationship. We interpreted quality of care as a distinct construct due to its non-specific nature, that is, it may represent experiences that are outside the scope of the patient-provider relationship. For instance, patients or caregivers may rate care as “very helpful” yet assess their relationships poorly. Similarly, satisfaction with care and continuity of care may signify technical rather than relational aspects of care. We, therefore, excluded these ambiguous markers of relationship quality.

## Results

### Study selection

**Figure 1.**
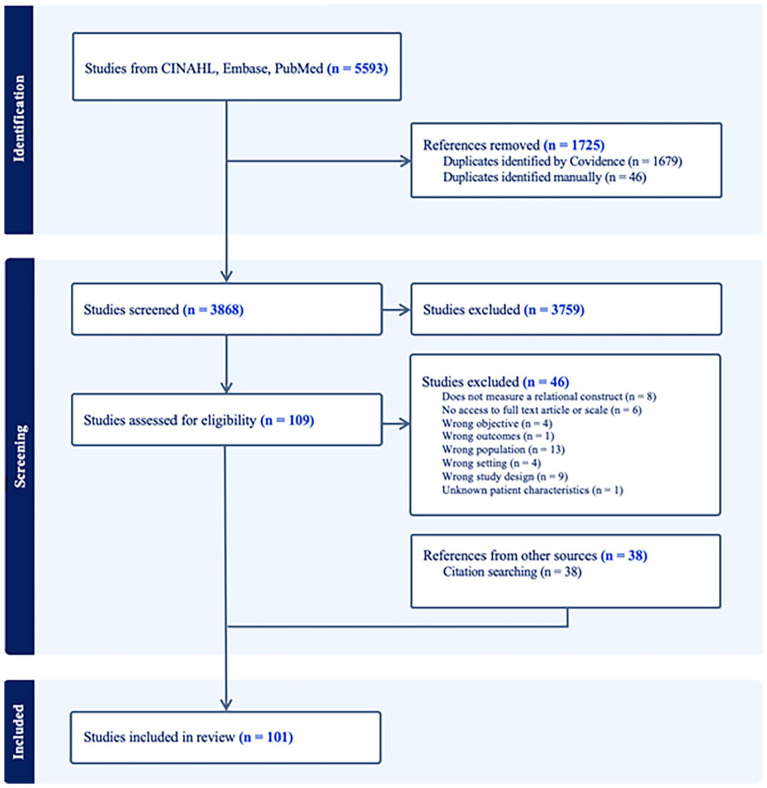
PRISMA-ScR diagram.

We included a total of 101 studies in this scoping review, which described findings from 47 unique measures (some of which had multiple versions). Figure 1 displays the PRISMA-ScR flow diagram.

### Study characteristics

Among the included studies, 43 were published in the United States. Studies were carried out across a range of settings in which people with serious illnesses are cared for, including inpatient and outpatient palliative care units, outpatient oncology care clinics, hospice care centers, community nursing homes offering hospice services, dialysis facilities, and other inpatient and outpatient facilities, some of which belonged to academic medical centers.

Valid measures (*n* = 47) reflected the experiences of patients and/or their surrogates (*n* = 26), or their caregivers/relatives (*n* = 15), or both (*n* = 6). Measures targeted a range of healthcare providers, including physicians, nursing staff (e.g. nurse aides, assistant nurses, nurse practitioners), technicians, social workers, psychologists and other palliative care team members.

Supplemental Materials 2A and 2B provide detailed summaries of the included studies (2A: patient/surrogate respondents; 2B: caregivers/relatives and other respondents).

### Characterization of relationship quality

We identified 47 measures of relationship quality validated in the context of patients with serious illness and subsequently applied a six-dimension model of relationship quality^26^ to consistently categorize them. We determined that 597 of 2238 items (26.7%) related to relationships. Most measures we identified (*n* = 46) contained items focused on engaging the patient as a whole person. Only nine measures contained items related to enabling patient management and navigation. Approximately half of the measures included items related to providers’ recognition of and response to emotions (*n* = 27) and promotion of shared decision-making (*n* = 23). An equal distribution of measures covered the elements of fostering a therapeutic alliance (*n* = 35) and promoting information exchange (*n* = 35).

We assessed the distribution of included items across the six elements of relationship quality (Supplemental Material 3). We found that 11 measures addressed five elements, 16 addressed four elements, 7 addressed three elements, 9 addressed two elements, and 1 addressed a single element. Only three instruments included items related to all six elements of relationship quality (CARE, CQ-index PC, and QPP).

### Engaging the patient as a whole person

Clarke et al.^26^ articulate the importance of seeing a patient as a person and not merely a disease by (1) adopting a biopsychosocial perspective, (2) respecting the individual, their needs, and their preferences, and (3) acknowledging the patient’s support systems and social contexts. Nearly all measures (46/47) include items that fit into one or several of these three sub-elements.

Items related to adopting a biopsychosocial perspective captured patients’ sense of feeling “completely cared for.”^27^ Questions assessed if a provider was interested in a patient as a “whole person”^28–40^ and “not just as a disease [or illness],”^39,41–44^ “patient,”^45^ or “someone with a medical problem,”^46,47^ and if “staff asked [patients] about how [their] disease affects other parts of [their] life”^28–35^ and “about what is happening in [their] daily life.”^48^

Items reflecting providers’ efforts to acknowledge the relational patient related to involving patients’ loved ones in their care and considering their wider social contexts and networks to understand them holistically. Measures assess whether doctors “listened to [families’] concerns about [patients’] medical treatment,”^49^ whether “the family’s wishes were respected in the selection of treatment”^50–53^ and “in deciding the place of referral to palliative care,”^54^ and whether “caregivers pay attention to [patients’] relatives.”^55^ Other items include “how well the involved ICU staff made [families] feel that [their] presence was appreciated”^56,57^ and the “willingness of ICU staff to answer [families’] questions.”^56,57^

Respecting the individual and their needs/preferences is a frequently articulated sub-element across these measures. Examples of items include: “Decisions about care and treatment are respected by the doctors and nurses,”^58^ doctors are “fully understanding [of patients’] concerns,”^36,37^ are “responsive to [their] needs or requests,”^59^ and “respect [the patient] as an individual.”^60^

### Promoting information exchange

A majority of measures (35/47) included items about information exchange between patients and/or other respondents and healthcare providers. Items representing this domain included satisfaction with information provision and frequency of provider communication.^28–35,40,49,56,57,61–95^ For instance, “Providers kept family members informed about [patient’s] condition.”^82,86^ Items also assess providers’ availability and willingness to speak with patients and caregivers/relatives in a timely manner.^28–35,39,41–44,56–59,61–64,66,68,70–72,74–78,81,85,91,92,96–98^ For instance, “How successfully did your healthcare provider communicate test results in a timely and sensitive manner?”^41–44^ and “How satisfied are you with willingness of ICU staff to answer your questions?”^91,92^ Others assess the way in which providers convey information: “How satisfied are you that the doctors explained things related to [patient’s illness] in a way [the patient or caregiver/relative] could understand” easily, clearly, and “in a straight-forward, honest manner?”^28–39,41–44,49,55–57,60,73,83–92,94–105^ Some items evaluate the consistency and quality of information provided.^28–35,55–57,73,83,84,86,91,92,96,97,106^ For instance, “How often did any doctor give confusing or contradictory information about [patient’s] medical condition?”^86^ and “Rate the overall quality of information provided to you by [doctors/nurses].”^56,57^

### Fostering therapeutic alliance

Therapeutic alliance consists of establishing and sustaining trust as well as sharing power with the patient.^26^ Measures (35/47) comprise some items that reflect provider actions/behaviors fostering therapeutic alliance with patients and/or other respondents. These items assess whether they had trust and confidence in their providers,^28–35,38,40–44,60,96,97,103^ whether providers’ presence was positive and supportive,^28–37,45,55–57,59,82–84,86–92,99,107–111^ whether providers spent enough time with patients,^28–35,39,41–44,55,93,99,103,112,113^ and how often providers were attentive and listened carefully.^28–35,38,41–45,50–53,55,94–97,112–116^ Items about trust include: “How satisfied are you with the level of trust and confidence you had in the doctors/nurses who looked after [you/your relative]?”^96,97^; “How often do you feel your [provider] is able to gain your trust?”^28–35^; and “How much do you trust your doctor?”^38^ Items about providers’ presence include: “Do your caregivers have a ‘warm’ attitude?”^55^; “Would you describe the clinician’s demeanor as [pleasant, compassionate, supportive, distant]?”^59^; “Providers were kind, caring, and respectful”^83,84^; and “How satisfied are you with the courtesy, respect, and compassion your family member was given?”^91,92^ Items about providers’ willingness to spend time include: “I felt I had enough time with my doctor”^93^; “I feel the doctor did not spend enough time with me”^39^; “Do your caregivers have enough time for you?”^55^; and “The care team spent the right amount of time with me.”^103^ Items about providers’ attentiveness include: “I have the feeling that the [doctors/nurses and assistant nurses] exhibited a sense of commitment”^87–90^; “How successfully did your doctor/healthcare provider listen attentively to you?”^41–44^; “How good is [Doctor] at giving you [his/her] full attention?”^94,95^; and “To what extent does your doctor pay close attention to what you are saying?”^38^

### Recognizing and responding to emotions

This element captures the importance of understanding and appropriately responding to emotional cues displayed by patients and/or their loved ones. Over half of the measures (27/47) articulated some form of emotional sensitivity shown by healthcare providers, as perceived by patients and/or other respondents. Measures assess the level and quality of emotional support provided to patients/families,^56,57,61–64,66,68,70–72,74–78,81–86,117–119^ whether support was given specifically in response to patients/families feeling anxious, stressed, or depressed,^28–35,50–53,55,86–90,96,97,99^ and whether providers expressed sensitivity and/or sympathy.^41–45,60,85,87–90,100–102^ For instance: “How often did your [provider] care about your emotional or psychological well-being?”^28–35^; “How satisfied are you with emotional support provided to [the patient/family members] by the palliative care team?”^61–64,66,68,70–72,74–78,81^; “Do you receive support when you feel [anxious/depressed]?”^55^; and “I receive the best possible help for [depression/anxiety].”^87–90^ Patient-reported items include: “I have the feeling that the [doctors/nurses and assistant nurses] showed sympathy when I was suffering”^87^ and “How successfully did your doctor or other healthcare provider express sensitivity, caring, and compassion for [your] situation?”^41–44^ Items reported by caregivers and others include: “The healthcare team was sensitive to my needs and feelings”^100–102^ and “How much support in dealing with your feelings about [patient’s] death did the doctors, nurses, and other professional staff. . .provide you?”^86^

### Sharing decision-making

Half of the measures in this review (23/47) included items related to shared decision-making. This element entails finding common ground with patients and involving them and/or their loved ones in care decisions. Items reflect whether patients feel included in discussions as a co-decider about their care and whether they participate to the extent they desire, ^28,31,32,36,37,40–44,50–53,55,58,60,65,67,69,73,80,87–90,93–97,118^ as well as whether their relatives could participate in decisions.^86,89^ For instance, “How successfully did your doctor/healthcare provider always involve you in decisions about your treatment?”^41–44^ and “Was there ever a decision made about [the patient’s] care without enough input from him/her or his/her family?”^86^ Items also assess whether caregivers and others feel fully involved in decision-making and are satisfied with their ability to share in their loved ones’ care.^33,56,57,61,63,64,68,70,71,74,76–79,81,91,92,96–98,118^ For instance, “How satisfied are you with the way the family is included in treatment and care decisions?”^61,63,64,68,70,71,74,76–78,81^

### Enabling self-management and patient navigation

Enabling self-management and patient navigation involves the promotion of patients’ autonomy through providers’ guidance around managing aspects of their care. Only nine of the 47 measures include items that characterize this element of relationship quality. Of all six relational elements, enabling patient management and navigation was least frequently articulated by the measures of relationship quality validated in the context of serious illness.

Items related to patient management in the measures we included assess whether providers “encourage [patients] to be as independent as possible,”^120–126^ “give [patients] freedom to plan [their] day,”^55,73^ and whether patients “had a sense of control about [their] treatment decisions.”^40,65,67,69,79,80^

Items related to patient navigation reflect whether “the care team helped [patients] understand what was important to [them],”^103^ and if “staff support [them] in coping”^120–126^ and “in living [the rest of their] life in a meaningful way.”^89,90^ Other items measure the extent to which patients feel that “interactions with the healthcare team helped [them] clarify their values and beliefs,” “talk about [their] concerns about the future and to be less frightened,” “better express and manage [their] feelings,” and “deal with changes in [their] relationships.”^127^

## Discussion

### Main findings of the study

We conducted a review to assess the scope of valid measures of relationship quality in the context of serious illness. We used an interpretive framework derived from literature on patient-centered care to examine and conceptualize the content of the measures in terms of six key elements of relationship quality.^26^ Overall, we found that the measures highlight the importance of several factors defining the quality of patient-provider relationships, including respect, trust, consistent and adequate information provision, supportive communication, and empathic engagement with and involvement of patients and/or their loved ones in their care. The most commonly appearing element across all measures was “Engaging with the patient as a whole person.” We found that an empirically derived framework for evaluating relationships provided a useful heuristic to categorize the measures.

We found variability in the distribution of elements and sub-elements of relationship quality across the measures included in this review. Although engaging the patient as a whole person was the most prevalent element, measures most frequently included items related to these sub-elements: “Respecting the individual, their needs, and preferences” and “Acknowledging the relational patient.” Measures which included items related to promoting information exchange focused on the sub-element of facilitating information exchange. Conversely, items related to fostering therapeutic alliance broadly assessed levels of trust. Measures less commonly assessed patients’ levels of involvement with decision-making and care self-management, which may be due to the focus of measurement tools on more observable indicators of relationship quality.

### What this study adds?

The survey items we identified assess key relational elements that align with current evidence on effective patient-provider relationships in the context of serious illness. One study defined an effective relationship in terms of being alongside the patient, maintaining cordiality, and fostering mutual understanding.^14^ In our review, we categorized these qualities as fostering a therapeutic alliance. In another study, patients and families ranked these as the most important end-of-life care domains: effective communication, shared decision-making, expert care, respectful and compassionate care, and trust/confidence in clinicians.^128^ While items related to “expert care” or provider skills were not among our inclusion criteria, these domains were commonly observed in the reviewed measures. Cancer patients have also described “good caring” as being respected, valued, listened to, given honest information, involved in decision-making, and as experiencing partnership in care.^129^

Communication mediates the quality of all patient-provider relationships. We found that measures commonly included items related to receiving holistic care and satisfactory information. These communication-related features represented two elements of relationship quality: “Engaging with the patient as a whole person” and “Promoting information exchange.” Cancer patients have articulated the importance of “being known” during meaningful encounters with providers.^130^ Studies of existential isolation implicate this feeling of being understood as a correlate of psychological outcomes and relationship quality has the potential to influence these outcomes.^131–133^ Accounts of being known were characterized by understanding a patient as a whole person, respecting patient autonomy and individual preferences, and building meaningful connections with patients.^130^ We also found measures assessing these indicators of being known across the relationship quality elements.

The role of communication in relationships as explored in this review may differ from other conceptions in the literature. One study suggests a need to develop more comprehensive measures of communication quality that represent patient and family perspectives within cancer care.^134^ They identified five communication domains that align with key elements of high-quality care: communicating information, interpersonal communication, communicating available supportive care services, communicating a transition in the objectives of care, and interprofessional communication. These were not expressed as distinct elements in our review; communication instead appeared as a concept related to all six elements of relationship quality.^26^ Patient-centered communication facilitates effective responses to emotional cues, collaborative engagement with patients, and better patient management of care. Honest communication fosters mutual trust between patients and providers. We classified items related to communicating information, including care objectives and the timing of provider communication, as promoting information exchange.

There are other factors that mediate the quality of relationships that would necessitate data collection to help contextualize findings from these measures (e.g. concordance of demographic or cultural factors). Currently, few instruments exist for measuring cultural aspects of care in settings of serious illness.^135,136^ Furthermore, writing about relationship-centered care gives insight into relational experiences that may not be captured by existing measures. For example, Matthews^137^ wrote of the transpersonal or spiritual dimension of care that is recognized in “moments of particular closeness during medical encounters. . .marked by a physiologic reaction, such as gooseflesh or a chill; by an immediacy of awareness of the patient’s situation (as if experiencing it from inside the patient’s world); by a sense of being part of a larger whole; and by a lingering feeling of joy, peacefulness, or awe.” None of the measures address these proximal relational experiences, despite their potential as indicators of meaningful connection.

This review may have important implications for clinical practice and future research on measure development. The measures we have identified may have utility in healthcare quality improvement initiatives and can inform clinical training programs aimed at improving patient-provider relationships. If it is true that relationships are a critical predictor of care quality, it may be important to consider the development of a single measure to assess relationship quality. *Of what constructs or questions might a single and parsimonious measure of patient-provider relationship quality consist?* We found significant overlap in the language used in measures. Table 3 draws from these measures to highlight questions and other items that may be useful candidates for a future relationship-focused instrument.

**Table 3.** Proposed items that assess elements of patient-provider relationship quality.^a^

Element	Items
Engaging the patient as a whole person	• How was your doctor at being interested in you as a whole person?^36,37^ • I felt this provider and team understood what is important to me in my life.^46,47^ • Do your caregivers pay attention to your relative(s)?^55^
Recognizing and responding to emotions	• [Doctor] understands my emotions, feelings, and concerns.^48^ • How satisfied are you with the emotional support you get from your healthcare team?^119^
Fostering therapeutic alliance	• How successfully did your doctor/healthcare provider gain your trust?^41–44^ • I felt this provider and team put my best interests first when making recommendations about my care.^46,47^
Promoting information exchange	• Do you feel that your doctor is informing you clearly and sincerely about your disease and about the therapy you are undergoing?^105^ • My questions were answered to my satisfaction.^93^
Sharing decision-making	• I participate as much as I want in the decisions about my care.^65,67,69,80^
Enabling self-management and patient navigation	• The care team helped me understand what was important to me.^103^ • How was your doctor at helping you take control?^36,37^

a We have reproduced the measures as they have been published but would recommend that future measures use more inclusive language that recognize the interprofessional nature of healthcare.

The measures we identified are cross-sectional and therefore may not be sensitive to expected changes in relationship quality over time. Responses, particularly to closed-ended questions, may carry some bias based on when surveys are completed. This phenomenon may lead to gaps in understanding the dynamic nature of relationship growth. A review of palliative care assessment tools identified one existing instrument related to continuity which authors categorized under “structure/process of care.”^136^ Although the framework we adopted includes continuity of care as an element of relationship quality,^26^ we distinguished care continuity from relational continuity and the evolving relationship dynamic.

### Strengths and limitations of the study

This review has limitations. First, our search strategy may have missed some relevant studies due to differences in available terminology representing relationship quality and due to limiting the date range in which studies were published. To maximize the inclusion of relevant literature, we consulted a librarian while iteratively refining the search strategy and manually searched reference lists of eligible studies. Second, we found that the United States was the most common study location; accordingly, the generalizability of our findings may be limited. Third, relationship quality can be conceptualized in multiple ways. To ensure a theoretically grounded and consistent review of measures, we employed an existing framework comprised of empirically derived domains.^26^ However, this framework is one of many and researchers applying a different framework may have evaluated the measures differently. Furthermore, our initial identification of measures as relationship-focused is subjective and may differ from the assessment of others. Last, we did not formally appraise the quality of studies included in this review. This limited our ability to rigorously assess the methodological validity of these studies. Arksey and O’Malley^18^ acknowledge this limitation of scoping reviews and suggest that this issue increases the quantity of data within reviews.

## Conclusion

We reviewed valid measures of patient-provider relationship quality in settings of serious illness and broadly defined the nature of these measures using a framework of relational elements of patient-centered care. Our findings can help researchers understand the degree to which and in what ways diverse patients, surrogates, and caregivers assess relationship quality. They also comprise candidate items for a single relationship-focused instrument, an example of which we proposed. Future research should explore cultural nuances to measuring patient-provider relationship quality. Our findings may also aid in the development of interventions for healthcare providers aiming to improve this critical mediator of care quality.

## Supplemental Material

sj-docx-1-pmj-10.1177_02692163251315304 – Supplemental material for Measuring the quality of patient-provider relationships in serious illness: A scoping review Supplemental material, sj-docx-1-pmj-10.1177_02692163251315304 for Measuring the quality of patient-provider relationships in serious illness: A scoping review by Karen Wassef, Kristine Ma, Brigitte N Durieux, Tyler L Brown, Joanna Paladino, Sally Thorne and Justin J Sanders in Palliative Medicine

sj-docx-2-pmj-10.1177_02692163251315304 – Supplemental material for Measuring the quality of patient-provider relationships in serious illness: A scoping review Supplemental material, sj-docx-2-pmj-10.1177_02692163251315304 for Measuring the quality of patient-provider relationships in serious illness: A scoping review by Karen Wassef, Kristine Ma, Brigitte N Durieux, Tyler L Brown, Joanna Paladino, Sally Thorne and Justin J Sanders in Palliative Medicine

sj-docx-3-pmj-10.1177_02692163251315304 – Supplemental material for Measuring the quality of patient-provider relationships in serious illness: A scoping review Supplemental material, sj-docx-3-pmj-10.1177_02692163251315304 for Measuring the quality of patient-provider relationships in serious illness: A scoping review by Karen Wassef, Kristine Ma, Brigitte N Durieux, Tyler L Brown, Joanna Paladino, Sally Thorne and Justin J Sanders in Palliative Medicine

sj-docx-4-pmj-10.1177_02692163251315304 – Supplemental material for Measuring the quality of patient-provider relationships in serious illness: A scoping review Supplemental material, sj-docx-4-pmj-10.1177_02692163251315304 for Measuring the quality of patient-provider relationships in serious illness: A scoping review by Karen Wassef, Kristine Ma, Brigitte N Durieux, Tyler L Brown, Joanna Paladino, Sally Thorne and Justin J Sanders in Palliative Medicine

sj-docx-5-pmj-10.1177_02692163251315304 – Supplemental material for Measuring the quality of patient-provider relationships in serious illness: A scoping review Supplemental material, sj-docx-5-pmj-10.1177_02692163251315304 for Measuring the quality of patient-provider relationships in serious illness: A scoping review by Karen Wassef, Kristine Ma, Brigitte N Durieux, Tyler L Brown, Joanna Paladino, Sally Thorne and Justin J Sanders in Palliative Medicine

sj-docx-6-pmj-10.1177_02692163251315304 – Supplemental material for Measuring the quality of patient-provider relationships in serious illness: A scoping review Supplemental material, sj-docx-6-pmj-10.1177_02692163251315304 for Measuring the quality of patient-provider relationships in serious illness: A scoping review by Karen Wassef, Kristine Ma, Brigitte N Durieux, Tyler L Brown, Joanna Paladino, Sally Thorne and Justin J Sanders in Palliative Medicine
