# Supplementary material for: Measuring the quality of patient-provider relationships in serious illness: A scoping review
Source: Palliat Med. 2025 Feb 6;39(3):332–45. doi: 10.1177/02692163251315304 (PMC11877987; doi:10.1177/02692163251315304)
Supplement: sj-docx-1-pmj-10.1177_02692163251315304 – Supplemental material for Measuring the quality of patient-provider relationships in serious illness: A scoping review [file sj-docx-1-pmj-10.1177_02692163251315304.docx]

|  | CONCEPT 1  **Serious illness** | CONCEPT 2  **Patient-provider relationships** | CONCEPT 3  **Measurement tools** | CONCEPT 4  **Validation** |
| --- | --- | --- | --- | --- |
| Keywords | “Terminal illness*” OR "terminally ill*" OR “life-limiting illness*” OR “serious illness*” OR "advanced illness*" | “Provider-patient” OR “patient-provider” OR “social worker patient” OR “patient-physician” OR “physician-patient” OR “nurse-patient” or “patient-nurse” OR "doctor-patient" OR "patient-doctor" OR "professional-patient" OR "patient-professional" | assess*[ti] OR measur*[ti] OR evaluat*[ti] OR scale*[ti] OR "instrument" OR "instruments" OR tool*[ti] OR "assessment tool*" OR "measurement tool*" OR questionnaire*[ti] OR survey*[ti] OR "patient reported" OR "quality indicator*" | valid* OR replica* OR accura* OR sensitiv* OR specific* OR responsiv* OR psychometric* |
|  | “Advanced cancer*” OR “metastatic cancer*” OR “end-stage renal disease*” OR “advanced heart failure*” OR “advanced COPD*” | ((support* OR communicat* OR connect* OR trust* OR "therapeutic alliance*" OR relationship* OR relational OR satisfaction) AND (physician* OR doctor* OR clinician* OR nurse* OR “social worker*” OR “health care provider*” OR “healthcare provider*” OR cardiologist* OR pulmonologist* OR oncologist* OR “health care”[ti] OR healthcare[ti] OR “clinical encounter*” OR "health personnel") AND patient*) |  |  |
|  | “Palliative care*” OR “hospice*” OR “terminal care*” OR “end-of-life care*” OR “compassion*”[ti] OR empathy[ti] | "Patient experience"[title/abstract:~3] OR "patients experiences"[title/abstract:~3] OR "clinical compassion" OR "clinician compassion" OR ((compassion*[ti] OR empathy[ti]) AND ("healthcare" OR "care")) |  |  |
| Subject headings | “Palliative Medicine”[Mesh] OR “Palliative Care”[Mesh] OR “Hospice Care”[Mesh] OR “Hospices”[Mesh] | “Physician-Patient Relations”[Mesh] OR “Nurse-Patient Relations”[Mesh] OR "Professional-Patient Relations"[Mesh:NoExp] OR “Therapeutic Alliance”[Mesh] | “Quality Indicators, Health Care”[Mesh:NoExp] OR  “Surveys and Questionnaires”[Mesh:NoExp] OR “validation study”[pt] OR “Patient Reported Outcome Measures”[Mesh:NoExp] | “Psychometrics”[Mesh] OR “validation study”[pt] OR “Validation Studies as Topic”[Mesh] |
| **Limits and filters:**  NOT (("Infant"[MeSH Terms] OR "Child"[MeSH Terms] OR "Adolescent"[MeSH Terms]) NOT "Adult"[MeSH Terms]) AND ((excludepreprints[Filter]) AND (1990:2023[pdat]) AND (english[Filter])) | | | | |
