# Supplementary material for: Measuring the quality of patient-provider relationships in serious illness: A scoping review
Source: Palliat Med. 2025 Feb 6;39(3):332–45. doi: 10.1177/02692163251315304 (PMC11877987; doi:10.1177/02692163251315304)
Supplement: sj-docx-2-pmj-10.1177_02692163251315304 – Supplemental material for Measuring the quality of patient-provider relationships in serious illness: A scoping review [file sj-docx-2-pmj-10.1177_02692163251315304.docx]

| **Table 2A. Patient and surrogate reported measures** | | | | | | | | | |
| --- | --- | --- | --- | --- | --- | --- | --- | --- | --- |
| **Measure name (number of items)** | | | | | | | | | |
| **Author, year (location)** | | | | | **Patient population and study setting** | **Respondent and provider** | | **Relational item(s) and measure format** | **Element of relationship quality** |
| ***1) Audit of Communication, Care Planning, and Documentation (ACCEPT) Questionnaire (24)*** | | | | | | | | | |
| Heyland, 2017 (Canada) | | | | | Inpatients from Canadian teaching hospitals with advanced medical diseases or who are ≥ 80 years old or any patient whose death is expected within the next six months. | Patients and family members assess the healthcare team (doctor, spiritual care, social work, nurse specialist). | | Unspecified measure format.  Goals-of-care discussion  Since the patient’s admission, a member of the healthcare team has asked the patient or substitute decision-maker or the patient’s family what is important to them as they consider healthcare decisions at this stage of the patient’s life (i.e., values, spiritual beliefs and other practices).  Since admission, a member of the healthcare team has asked the patient or his or her family if they had any questions or needed things clarified regarding the patient’s overall goals of care.  Since admission, a member of the healthcare team has given the patient the opportunity to express his or her fears or discuss what concerns him or her.  Since admission, a member of the healthcare team has asked the patient what treatments he or she prefers to have or not have if he or she develops a life-threatening illness.  Since admission, the patient and family have been offered support from the allied healthcare team as needed. | 1  1, 3, 4  1  1  1, 2 |
| ***2) Brief Hospice Inventory (BHI) (17)*** | | | | | | | | | |
| Guo, 2001 (USA) | | | | | Patients receiving home-based hospice care in the U.S (Arizona and Utah). | Patients (and nurse caregiver if assistance needed) assess their general experience around hospice care. | | On a 0-10 numeric rating scale…  Over the past few days I have felt: Completely cared for (0)…Not cared for at all (10) | 1, 3 |
| ***3) Canadian Health Care Evaluation Project (CANHELP) Questionnaire*** | | | | | | | | | |
| CANHELP (patient version: 38) | Heyland, 2010 (Canada) | | | | Patients from inpatient and outpatient facilities and home care programs in Canada who are (a) > 55 years old with COPD, CHF, cirrhosis and/or metastatic cancer or (b) ≥ 80 years old and admitted to a hospital or (c) enrolled in a home care program using long-term oxygen therapy and have COPD, CHF, or metastatic cancer. | Patients and caregivers assess doctors, nurses, and other healthcare professionals. | | Degree of satisfaction is rated from 1 (“Not at all satisfied”) to 5 (“Completely satisfied”).  How satisfied are you (with respect to the last month of care)…  Relationship with the doctors  that your doctor(s) took a personal interest in you?  that your doctor(s) were available when you needed them?  with the level of trust and confidence you had in the doctor(s)?  Illness management  with the level of trust and confidence you had in the nurses who looked after you?  that the doctors and nurses were compassionate and supportive?  that you were treated in a manner that preserved your sense of dignity?  that the emotional problems you had were adequately assessed and controlled?  that the care and treatment you received was consistent with your wishes?  Communication  that the doctor(s) explained things relating to your illness in a straight-forward, honest manner?  that the doctor(s) explained things related to your illness in a way you could understand?  that you received consistent information about your condition from all doctors/nurses?  that the doctor(s) listened to what you had to say?  that you received updates about your condition, treatments, test results, etc. in a timely manner?  Decision making  with discussions with your doctor(s) about where you would be cared for if you were to get worse?  with discussions with your doctor(s) about the use of life sustaining technologies?  with your role in decision making regarding your medical care? | 1  3  3  3  3  1  2  1  4  4  4  1, 3  4  4  4  5 |
| CANHELP-Lite (patient version: 21) | Heyland, 2013 (Canada) | | | |  |  |  | Degree of satisfaction is rated from 1 (“Not at all satisfied”) to 5 (“Completely satisfied”).  How satisfied are you (with respect to the last month of care)…  Relationship with the doctors  that the doctor(s) took a personal interest in you?  that the doctor(s) were available when you needed them?  with the level of trust and confidence you had in the doctor(s) who looked after you?  Illness management  that you were treated by those doctors, nurses, and other healthcare professionals in a manner that preserved your sense of dignity?  that the emotional problems you had were adequately assessed and controlled?  that the care and treatment you received was consistent with your wishes?  Communication  that the doctor(s) explained things relating to your illness in a straight-forward, honest manner?  that you received consistent information about your condition from all the doctors and nurses looking after you?  that the doctor(s) listened to what you had to say?  Decision making  with discussions with your doctor(s) about where you would be cared for if your condition worsened?  with discussions with your doctor(s) about the use of life sustaining technologies?  with your role in decision-making regarding your medical care? | 1  3  3  1  2  1  4  4  1, 3  4  4  5 |
| ***4) Care Evaluation Scale (CES)*** | | | | | | | | | |
| CES – Patient Version (CES-P) (23) | Miyashita, 2014 (Japan) | | | | Cancer patients aged ≥ 20 years receiving treatment in an inpatient palliative care unit or an oncology ward or outpatient clinic of a university medical center in Japan. | Patients assess physicians and nurses. | | Necessity for improvement for each item is rated on a six-point Likert scale from 1 (“Improvement is not necessary”) to 6 (“Improvement is highly necessary”).  Psycho-existential care  Doctors and nurses pay attention to relieving your concerns and worries.  Doctors and nurses take appropriate measures when you become depressed.  Doctors and nurses try so that your hope would be accomplished.  Help with decision making by physician  The doctors give sufficient explanations to you about your present condition and the details of your medical treatment.  The doctors give sufficient explanations to you about the expected outcome.  Consideration is given so that you can participate in the selection of treatment.  Availability  Admission (use) is in accordance with the wishes of you and your family. | 1  2  2  4  4  5  1 |
| Cancer CES (CCES) (35) | Masukawa, 2020 (Japan) | | | | Patients aged ≥ 20 years with a diagnosis of a solid tumor and an admission to a hospital in Japan within the last two years. | Patients assess physicians and nurses. | | Six-point Likert scale from 1 (“Absolutely disagree”) to 6 (“Absolutely agree”).  Relationship with physician  Doctors listen to you carefully.  Doctors understand you adequately.    Doctors develop a good relationship with you.  Relationship with nurse  Nurses listen to you carefully.  Nurses understand you adequately.  Nurses develop a good relationship with you.  Psycho-existential care  Consideration was paid to relieving your concerns and worries.  The staff took appropriate measures when you became depressed.  The staff tried to give you hope.  **Help with decision-making for patients**  Consideration was given so that you could participate in the selection of treatment.  The doctors gave you sufficient explanations about the expected outcome.  The doctors gave you sufficient explanations about your present condition and details of your medical treatment.  Admission  Admission (use) was in accordance with the wishes of the patient and family. | 1, 3  3  3  1, 3  3  3  1  2  2  5  4  4  1 |
| ***5) Chronic Cancer Experiences Questionnaire (CCEQ) (75)*** | | | | | | | | | |
| Harley, 2019 (UK) | | | | | Patients with chronic breast, colorectal/gastrointestinal, renal, prostate or gynecological disease attending outpatient oncology clinics at a cancer unit in England for treatment, review or follow-up assessments. | Patients assess general practitioner, nurse, or other medical staff. | | Items are rated on a five-point Likert scale (“Strongly agree” to “Strongly disagree”).  Coordination of care  The staff at the hospital are friendly and make me feel at ease.  I feel there is enough time to ask questions when I come to clinic.  The doctors are very open and will tell you anything you need to know.  I am content with the information I have received about my diagnosis.  I am content with the information I have received about my prognosis.  Making treatment decisions  I am given the opportunity to discuss my treatment plan with the doctors.  My decisions about care and treatment are respected by the doctors and nurses.  If I have questions about new treatments, my doctor is happy to discuss these with me.  Key worker  At times I have felt abandoned by the medical staff. | 3  4  4  4  4  5  1  4  3 |
| ***6) Clinical Evaluation Questionnaire (CEQ) (7)*** | | | | | | | | | |
| de Vries, 2022 (Canada) | | | | | Patients with advanced cancer (stage III or IV cancer with a prognosis of 12–18 months) at a teaching hospital in Canada. | Patients assess healthcare providers. | | Items are rated on perceived helpfulness of the therapeutic interactions on a five-point Likert scale, ranging from 0 (“Not at all”) to 4 (“Very much”), with a “Not applicable” option.  To what extent have your interactions with the healthcare team helped you to:  Freely discuss my concerns about cancer and my treatment options.  Talk and feel understood about how cancer has affected my life.  Deal with changes in my relationships as a result of cancer.  Explore better ways to communicate with my healthcare team, my family and others.  Clarify my values and beliefs.  Talk about my concerns about the future and to be less frightened.  Better express and manage feelings. | 5  1  6  6  6  6  6 |
| *7) Compassion Measure (5)* | | | | | | | | | |
| Roberts, 2019 (USA) | | | | | Patients who had an outpatient clinic visit in an academic healthcare system in the U.S (New Jersey). | Patients assess physicians, advanced nurse  practitioners, or physician assistants from >15 specialties, including hematology/oncology, pulmonary, nephrology, etc. | | Item responses range from 1 (“Never”) to 4 (“Always”).  How often do you feel your provider… (Roberts, 2019) / During this hospital stay, how often do you feel your [doctors/nurses]… (Roberts, 2021)  cares/cared about your emotional or psychological well-being?  is/were interested in you as a whole person?  is/were considerate of your personal needs?  is/were able to gain your trust?  shows/showed you care and compassion? | 1, 2  1  1  3  3 |
| Roberts, 2021 (USA)* | | | | | Patients who had at least one overnight stay in hospitals within an acute hospital system in the U.S. | Patients assess physicians and nurses. | |  |  |
| ***8) Consumer Assessment of Healthcare Providers and Systems (CAHPS®)*** | | | | | | | | | |
| CAHPS In-Center Hemodialysis Survey (ICH-CAHPS) (35) | Weidmer, 2014 (USA) | | | | Patients with chronic kidney disease who received hemodialysis for ≥ three months at dialysis facilities in the U.S. | Patients assess nephrologists and dialysis center staff, including nurses, technicians, dietitians, and social workers. | | Item responses range from 1 (“Never”) to 4 (“Always”) or “Yes”/“No” or from 0 (“Worst”) to 10 (“Best”) for global ratings.  Your Kidney Doctors/The Dialysis Center Staff  In the last 3 months, how often did your kidney doctors/dialysis center staff…  listen carefully to you?  explain things in a way that was easy to understand?  show respect for what you had to say?  spend enough time with you?  really cared about you as a person?  make you as comfortable as possible during dialysis?  Did you feel comfortable asking the dialysis center staff everything you wanted about dialysis care?  *[Additional item in Weidmer, 2014; Wood, 2014]* Has anyone on the dialysis center staff asked you about how your kidney disease affects other parts of your life?  How often did dialysis center staff explain blood test results in a way that was easy to understand?  How often did dialysis center staff behave in a professional manner?  Treatment/Providing information to patients  In the last [3 or 12] months…  Did either your kidney doctors or dialysis center staff talk to you as much as you wanted about which treatment is right for you?  Were you as involved as much as you wanted in choosing the treatment for kidney disease that is right for you?  *[Additional items in Wood, 2014]* Were you ever unhappy with the care you received at the dialysis center or from your kidney doctors? [If yes] How often were you satisfied with the way they handled these problems?  How often did dialysis center staff check you as closely as you wanted while you were on the dialysis machine?  Global ratings *[Additional items in Weidmer, 2014; Wood, 2014]*  What number would you use to rate the kidney doctors you have now?  What number would you use to rate your dialysis center staff? | 1, 3  4  1  3  1  1  3    1    4  3  4, 5  5    3    3  3  3  4  1, 3  1  3 |
| CAHPS-ICH (58) | Wood, 2014 (USA) | | | |  |  |  |  |  |
| ICH-CAHPS (32) | Setodji, 2019 (USA) | | | |  |  |  |  |  |
| CAHPS (5) | Hays, 2023 (USA) | | | | Patients with serious illness (cancer, heart failure, COPD, end-stage liver disease, end-stage renal disease, or amyotrophic lateral sclerosis) receiving outpatient care from physicians in primary care clinics in the U.S (California). | Patients assess their personal doctor (doctor, nurse practitioner, or any member that patient sees for regular medical care). | | Item responses range from 1 (“Never”) to 4 (“Always”).  How often did this provider…  explain things in a way that was easy to understand?  listen carefully to you?  show respect for what you had to say?  spend enough time with you? |  |
| ***9) Consultation and Relational Empathy (CARE)*** | | | | | | | | | |
| CARE_R_-measure (9) | Wirtz, 2011 (Germany) | | | | Inpatients aged ≤ 75 years with bronchial, oesophageal, colorectal, breast, prostate or skin cancer at a university clinic in Germany. | Patients assess physicians. | | Five-point Likert scale ranging from “Poor” to “Excellent” with a “Not applicable” option.  How was the doctor at…/ How did the physician primarily responsible for your treatment…behave in general (Wirtz, 2011)?  Making you feel at ease.  Letting you tell your story.  Really listening.  Being interested in you as a whole person.  Fully understanding your concerns.  Showing care and compassion.  Being positive.  Explaining things clearly.  Helping you take control.  Making a plan of action with you. | 2  1, 3  1, 3    1  1  3  3  4  6  5 |
| Korean CARE Measure (10) | Park, 2022 (Korea)* | | | | Patients with angina, myocardial infarction, valvular heart disease, heart failure, arrhythmia, hypertension, hyperlipidemia, or diabetes mellitus at a cardiology outpatient clinic (secondary and tertiary care hospitals) or family medicine outpatient clinic (tertiary care hospital) in Korea. |  |  |  |  |
| ***10) Consumer Quality Index Palliative Care (CQ-index PC) (88)*** | | | | | | | | | |
| Claessen, 2012 (Netherlands) | | | | | Patients with a life expectancy of six months or less and/or who were receiving palliative treatment at high-care hospices, hospitals, nursing home, home for the elderly, home care organizations, or mental healthcare organization. | Patients assess medical caregivers. | | Four-point scale from (“Never”) to (“Always”) or “Yes”/”No” options.  Care for psychosocial well-being  Do you receive support when you feel anxious?  Do you receive support when you feel depressed?  Are your caregivers polite to you?  Do your caregivers listen carefully to you?  Do your caregivers have enough time for you?  Do your caregivers take you seriously?  Do your caregivers show interest in your personal situation?  Do your caregivers have a ‘warm’ attitude?  Do you have the opportunity to talk to your caregivers about how you are feeling?  Do your caregivers pay attention to your relative(s)?  Care for spiritual well-being  Do your caregivers respect your life stance?  Respecting independence  Do your caregivers give you the chance to plan your own day?  Are you involved in decisions about your care?  Do your caregivers take your personal wishes into account?  Information  Do your caregivers explain things to you in a way you could understand?  Do your caregivers give you contradictory information?  Expertise of caregivers  Do you receive help in good time when you are in need of care?  Are you offered help in good time in acute situations? | 2  2  3  1, 3  3  1, 3  1  3  3  1  1  5, 6  5  1  4  4  3  3 |
| ***11) Eight-Factor Model of Patients’ Experiences: What Terminally Ill Patients Care About (51)*** | | | | | | | | | |
| Emanuel, 2000 (USA) | | | | | Patients with any illness (except HIV/AIDS) with a six month prognosis in hospitals in the U.S. | Patients assess doctors and nurses. | | Items are rated on a numeric scale from 0 to 10.  Patient-clinician relationship  Your doctor/nurse respects you as an individual.  You feel that your doctor/nurse will help you through the medical system to get the medical care you need.  You feel that you are able to participate in decisions about your care.  You feel that your doctor/nurse listens to what you have to say about your illness or medical treatment.  You have complete trust in your doctor/nurse.  Your primary doctor tells you bad news in a sensitive and caring manner.  Clinician communication  Your doctor/nurse has given you clear information about what to expect regarding your illness and outlook.  Your doctor/nurse has given you clear information about the risks and side effects of your treatment.  Your doctor/nurse has given you a clear explanation of your treatment alternatives. | 1  1, 3  5  1, 3  3  2  4  4  4 |
| ***12) Family Satisfaction with End-of-Life Care (FAMCARE) Scale*** | | | | | | | | | |
| FAMCARE-P13 (13) | Lo, 2009 (Canada) | | | | Outpatients at a Canadian oncology clinic with locally advanced pancreatic cancer or metastatic gastrointestinal, genitourinary, breast, lung or gynecological cancer, Eastern Cooperative Oncology Group performance status from 0 to 2, and a prognosis of six months to two years. | Patients assess health professionals, including doctors and nurses. | | Five-point scale where items are rated from 1 (“Very dissatisfied”) to 5 (“Very satisfied”).  How satisfied are you with:  Information given about how to manage pain.  The availability of nurses to answer your questions.  ﻿﻿﻿Information provided about your prognosis.  Information given about your tests.  The availability of doctors to answer your questions.  Information given about side effects.  The way the family is included in treatment and care decisions.  *[Additional items in FAMCARE-P16]*  Doctor’s attention to your description of symptoms.  The availability of the doctor to your family. | 4  3, 4  4  4  3, 4  4  1, 5  1, 4  1 |
|  | Parpa, 2017 (Greece) | | | | Patients with cancer treated in a university outpatient palliative care unit in Greece. |  |  |  |  |
| FAMCARE-P16 (16) | Lo, 2009 (Canada) | | | | Patients with metastatic cancer in outpatient oncology clinics at a hospital in Canada. |  |  |  |  |
| ***13) Feeling Heard and Understood (4)*** | | | | | | | | | |
| Edelen, 2022 (USA) | | | | | Patients receiving ambulatory clinic-based palliative care in the U.S. | Patients assess providers including physicians, advanced practice nurses, therapists, and clinical psychologists. | | Item responses range from 1 (“Completely true”) to 5 (“Not at all true”).  I felt heard and understood by this provider and team.  I felt this provider and team put my best interests first when making recommendations about my care.  I felt this provider and team saw me as a person, not just someone with a medical problem.  I felt this provider and team understood what is important to me in my life. | 1  1, 3  1  1 |
| Walling, 2023 (USA) | | | | |  |  |  |  |  |
| ***14) Hospital Quality of Life Index Taiwanese Version (HQOL-T) (28)*** | | | | | | | | | |
| Longcoy, 2023 (Taiwan) | | | | | Patients with advanced cancer from palliative inpatient units of a veterans general hospital in Taiwan. | Patients assess healthcare providers. | | Item responses range from 0 (“Very dissatisfied”) to 10 (“Very satisfied”).  How satisfied are you with the…  emotional support you get from your healthcare team?  spiritual support you get from your healthcare team? | 2  1 |
| ***15) The Human Connection (THC) Scale (16)*** | | | | | | | | | |
| Mack, 2009 (USA) | | | | | Patients aged ≥ 20 years from cancer centers and clinics in the U.S who have gastrointestinal or thoracic cancer and who experienced failed first-line or second-line chemotherapy. | Patients assess physicians. | | Item responses range from 1 to 4: “Never” to “At every office visit”, or “Not at all” to “To a large extent”, or “Not at all” to “A great deal”, or “Never” to “Always”, or “Not concerned” to “Extremely concerned.”  How often would you say your doctor takes the time to listen to your concerns?  To what extent does your doctor pay close attention to what you are saying?  To what extent do you think your doctor sees you as a whole person?  How much do you like your doctor?  How much do you trust your doctor?  How thorough is your doctor?  How much do you respect your doctor?  How much do you feel your doctor cares about you?  How much of the time would you say your doctor is honest with you?  To what extent do you feel comfortable asking your doctor questions?  How often do you understand your doctor’s explanations and suggestions?  How often does your doctor ask how family members are coping with your illness?  How often does your doctor offer hope?  How often does your doctor ask how you are coping with cancer?  How concerned do you think your doctor is about your quality of life? | 1, 3  1, 3  1  3  3  3  3  1, 3  4  4  4  1  2  3  1 |
| ***16) ICU-RESPECT (10)*** | | | | | | | | | |
| Geller, 2016 (USA) | | | | | Medical and surgical patients in ICUs within the Johns Hopkins Health System in the U.S. | Patients and/or family or friends assess the care team. | | Item responses are dichotomized into “Never/Most of the time” and “All of the time.”  Members of the care team treated [me/my loved one] with courtesy.  Members of the care team made an effort to understand what matters to [me/my loved one] most.  Members of the care team were attentive to [my/my loved one's] requests.  [I/My loved one] felt that the care team really listened to [me/him or her].  Members of the care team made efforts to know [me/my loved one] as a unique individual.  Members of the care team treated [me/my loved one] as their equal.  Members of the care team treated [me/my loved one] the way they would like to be treated if they were the patient. | 1, 3  1  1  1, 3  1  1, 3  1, 3 |
| Geller, 2018 (USA) | | | | | Patients who have been in a medical-surgical ICU in a west coast academic medical center in the U.S for at least three days. | Patients (or family members if patients could not complete the survey) assess the care team. | |  |  |
| ***17) Jefferson Scale of Patient’s Perceptions of Physician Empathy (JSPPPE) (6)*** | | | | | | | | | |
| Borracci, 2017 (Argentina) | | | | | Outpatients from an urban public hospital, private medical center, and a suburban clinic in Argentina. | Patients assess physicians. | | Items are rated on a five-point Likert scale from 1 (“Strongly Disagree”) to 5 (“Strongly agree”).  Dr. X:  Understands my emotions, feelings, and concerns.  Seems concerned about me and my family.  Can view things from my perspective.  Asks about what is happening in my daily life.  Is an understanding doctor.  Devotes enough time to me. | 1, 2  1  1  1  3  3 |
| ***18) Kidney Diseases Quality of Life (KDQOL) Instrument*** | | | | | | | | | |
| KDQOL (134) | | | Hays, 1994 (USA) | | Patients with kidney disease receiving dialysis at outpatient dialysis centers in the U.S. (California). | Patients assess dialysis staff. | | Item responses range from 1 (“Very poor) to 7 (“The best”) or 1 (“Definitely true”) to 5 (“Definitely false”) / Satisfaction is rated from “Extremely satisfied” to “Extremely dissatisfied” (Green, 2001).  Satisfaction With Care  Think about the care you receive for kidney disease. In terms of your satisfaction, how would you rate the friendliness and interest shown in you as a person?  How true or false is each of the following statements?  Dialysis staff encourage me to be as independent as possible.  Dialysis staff support me in coping with my kidney disease. | 1  6  6 |
| Japanese version of KDQOL (43) | | | Green, 2001 (Japan) | | Patients receiving dialysis for three or more months in dialysis centers in Japan. |  |  |  |  |
| KDQOL-Short Form (SF) (80) | | | Korevaar, 2002 (Netherlands) | | Patients with end-stage renal disease in centers for chronic dialysis treatment in the Netherlands. |  |  |  |  |
| Korean version of KDQOL-SF (80) | | | Park, 2007 (Korea) | | Patients receiving hemodialysis or continuous ambulatory peritoneal dialysis in university dialysis centers in Korea. |  |  |  |  |
| Italian version of KDQOL-SF version 1.3 (79) | | | Klersy, 2007 (Italy) | | Patients enrolled in referral centers in Italy and receiving dialysis or hospitalized for chronic kidney disease. |  |  |  |  |
| Iranian version of KDQOL-SF version 1.3 (79) | | | Pakpour, 2010 (Iran) | | Iranian patients receiving dialysis treatment for more than three months at dialysis centers in Iran. |  |  |  |  |
| Urdu version of KDQOL-SF-36 (36) | | | Anees, 2016 (Pakistan) | | Patients with end-stage renal disease on maintenance hemodialysis (MHD) for more than three months at the nephrology department of a hospital in Pakistan. |  |  |  |  |
| ***19) LifeCourse Experience Tool (21)*** | | | | | | | | | |
| Fernstrom, 2016 (USA)* | | | | | Patients from hospitals or clinics in the U.S (Minnesota) with serious chronic illness in later life. | Patients assess the care team, including physicians, nurses, aides,  care guides, social workers, chaplains, etc. | | Four-point, frequency-based adjectival scale from 1(“Never”) to 4 (“Always”), except for items related to patients’ goals for their care, which used a Likert scale with agreement-based responses from 1 (“Strongly Disagree”) to 4 (“Strongly Agree”).  The care team helped me make a choice about my care when I had one.  The care team kept my wishes at the center of my care.  The care team helped me understand all of my options when I had a choice about my care.  The care team respected me.  The care team helped me understand what was important to me.  I trusted my care team.  The care team spent the right amount of time with me.  I received easy to understand information from the care team in response to my questions.  The care team did everything they could to help with my problem or physical symptom.  I was able to get in touch with someone on my care team when needed.  The care team knew my personal circumstances or situation.  I had to repeat myself when telling the care team about my life.  I had to repeat myself when telling the care team about my medical condition.  I had to repeat myself when telling the care team about what was important to me.  I had unanswered questions about how my illness affected my everyday life.  I had unanswered questions about how my illness affected my health.  My goals of care include what is important to me. | 5  1  4  1  6  3  3  4  1  3  1  1  1  1  4  4  1, 5 |
| ***20) Patient Dignity Inventory (PDI) (25)*** | | | | | | | | | |
| English version (PDI) | | Chochinov, 2008 (Australia and Canada) | | | Patients enrolled in Australian and Canadian palliative care programs and who have a life expectancy of less than six months. | Patients assess healthcare providers. | | Five-point scale from 1 (“Not a problem”) to 5 (“An overwhelming problem”).  Indicate how much of a problem or concern these have been for you within the last few days:  Not feeling supported by my healthcare providers.  Not being treated with respect or understanding by others. | 1, 3  1 |
| Italian version of PDI (DiPO) | | Ripamonti, 2012 (Italy) | | | Outpatients with a Karnofsky performance score ≥70 and a life expectancy of > six months receiving oncological therapy in four units in Italy: Supportive care in cancer unit (National Cancer Institute), oncological unit or hematology division in university hospitals, or Center of Oncological Rehabilitation. |  |  |  |  |
| German version (PDI-G) | | Sautier, 2013 (Germany) | | | Patients with cancer receiving curative and palliative treatment in a university cancer center in Germany. |  |  |  |  |
| Spanish version (PDI-s) | | Rullán, 2015 (Spain) | | | Patients with advanced cancer and a Karnofsky performance score > 30 from the Department of Oncology and Palliative Medicine of a university clinic in Spain. |  |  |  |  |
| Czech version (PDI-CZ) | | Kisvetrová, 2017 (Czech Republic) | | | Patients from institutions providing palliative or long-term care in Czech Republic (oncology and geriatric departments, long-term care facilities, and nursing homes) who have terminal cancer or severe chronic noncancer illness or severe geriatric frailty and a life expectancy of > six months. |  |  |  |  |
| ***21) Patient-Physician Relationship Index (PPRI) (8)*** | | | | | | | | | |
| Ostacoli, 2007 (Italy) | | | | | Patients with metastatic breast, colorectal or lung cancer admitted for chemotherapy at departments of Medical Oncology in Italy. | Patients assess the medical team, including doctors and nursing personnel. | | Five-point Likert scale from 1 (“Not at all”) to 5 (“Very much”).  Do you feel that your doctor is informing you clearly and sincerely about your disease and about the therapy you are undergoing?  Do you feel comfortable when you ask him/her questions and require explanations?  Do you feel that he/she is treating you as a human being?  Is the relationship with your doctor important for your emotional well-being?  Do you think that the nursing personnel is efficient and helpful?  Do you feel that everything possible is being done for you? | 4  3  1  2  3  3 |
| ***22) Patient Satisfaction with Cancer Care for Spanish speakers (PSCC-Sp) (18)*** | | | | | | | | | |
| Jean-Pierre, 2012 (USA) | | | | | Spanish speaking patients with cancer screening abnormalities or diagnosed cancer (breast, cervical, colorectal or prostate cancer) receiving cancer-related care from clinics and hospitals affiliated with the Patient Navigation Research Program in the U.S. | Patients assess doctors and specialists. | | Five-point Likert scale from 1 (“Strongly agree”) to 5 (“Strongly disagree”).  I felt that my health concerns were understood.  I felt that I was treated with courtesy and respect.  I felt included in decisions about my health.  I felt encouraged to talk about my personal health concerns.  I felt I had enough time with my doctor.  My questions were answered to my satisfaction.  I was able to get the advice I needed about my health issues.  The doctors seemed to communicate well about my care. | 1  1  5  3  3  4  4  4 |
| ***23) Princess Margaret Hospital Patient Satisfaction with Doctor Questionnaire (PMH/PSQ-MD) (29)*** | | | | | | | | | |
| Loblaw, 2003 (Canada) | | | | | Oncology patients from outpatient clinics of a Canadian tertiary care center. | Patients assess doctors. | | Four-point Likert scale from 1 (“Strongly agree”) to 5 (“Strongly disagree”) with a “Does not apply” option.  The doctor did not take my problems very seriously.  The doctor considered my individual needs when treating my condition.  The doctor did not give me all the information I thought I should have been given.  The doctor went straight to my medical problem without greeting me first.  The doctor used words I did not understand.    I feel the doctor did not spend enough time with me.    It seemed to me that the doctor was not really interested in my emotional well-being.    I really felt understood by my doctor.    After my last visit with my doctor, I feel much better about my concerns.    The doctor was not friendly to me.    I understand my illness much better after seeing this doctor.    This doctor was interested in me as a person and not just my illness.  I would not recommend this doctor to a friend.  The doctor seemed to brush off my questions.  The doctor should have told me more about how to care for my condition.  The doctor told me to call back if I had any questions or problems.  I felt the doctor was being honest with me.  It seemed to me that the doctor was not really interested in my physical well-being.  The doctor should have shown more interest. | 1  1  4  1, 3  4  3  2  1  1  3  4  1  3  3  4  4  4  1  1 |
| ***24) Problem Scores (45)*** | | | | | | | | | |
| Teno, 2009 (USA) | | | | | Patients from oncologist offices in the U.S (New Hampshire, Connecticut, Rhode Island) who are diagnosed with advanced cancer. | Patients assess cancer care providers and/or other physicians involved in care (primary care, surgeon, radiation oncologist). | | Item responses include dichotomous “Yes” or “No” categories or range from “Always” to “Never” or from “Less than was needed” to “Just the right amount.”  Communication at the time of diagnosis  Was your prognosis explained to you in a way that you could understand?  Was your prognosis presented to you in a sensitive manner?  How much emotional support did you receive from your oncologist or cancer care providers when your prognosis was discussed with you?  Were you allowed to ask as many questions as you wanted about the prognosis of your cancer?  How often have you been given confusing or contradictory information about your prognosis from your oncologist or other cancer care providers?  How often does your oncologist or cancer care provider explain your test results in a way that you can understand?  How often do you want more information about your test results than you receive from your cancer care providers?  How often have you been given confusing or contradictory information about your test results from your cancer care providers and/or other physicians involved in your care?  Do you feel that your oncologist or cancer care providers listen to your concerns about your treatment plan?  How often do your cancer care providers allow your family/friends to ask as many questions as they want about your treatment plan?  How often do you feel that your oncologist or cancer care providers are not paying enough attention to your care?  How often do you feel that you and your family are left on your own to make sure that the right things get done for your cancer care?  Communication at the time of treatment decision making  How often do you feel that your cancer care providers listen to your concerns about the possible side effects of chemotherapy and/or radiation treatments?  How often do you feel that you and your family are left on your own to make sure that the right things get done for your cancer care?  How often do your cancer care providers explain what they are doing to do in a way that you can understand?  Cancer patient experience of treatments  How often do your cancer care providers do everything they can to promote your comfort during treatment?  How often are your cancer care providers available to speak with you when you have a concern about your chemotherapy?  How often is everything done to make you as comfortable as possible while you are receiving your chemotherapy treatment?  How much support do you get from the staff during chemotherapy treatments?  How often do the staff promote your dignity during chemotherapy treatments?  Overall, how often have you been treated with respect by your cancer care providers? | 4  2  2  3, 4  4  4  4  4  1  1, 4  1  1  1  1  4  1  1, 3  1  1, 3  1  1 |
| ***25) Quality Care Questionnaire-End of Life (QCQ-EOL) (16)*** | | | | | | | | | |
| Yun, 2006 (Korea) | | | | | Patients diagnosed with terminal cancer in conventional care facilities and hospices in Korea. | Patients assess healthcare professionals. | | Response items range from 1 (“Not a lot”) to 4 (“Very much”).  Have you a good relationship with your healthcare providers?  Have healthcare providers given you clear explanations about your health status? | 3  4 |
| ***26) Quality from the Patient’s Perspective (QPP)*** | | | | | | | | | |
| Revised QPP (64) | | | | Larsson, 1998 (Sweden) | Patients hospitalized for at least two days in gynaecological, medical, orthopedic, and surgical departments of a Swedish hospital. | Patients assess doctors, nurses, and assistant nurses. | | Item responses range from 1 (“Do not agree at all”) to 4 (“Fully agree”).  I have…  Medical-technical competence  The help I wanted/needed:   - to take care of my personal hygiene - in relation to using the toilet - to sit and lie comfortably - in relation to my meals   Identity-oriented approach  Satisfactory information regarding   - Medical examinations and tests, so that I understood their relevance as well as how they would be implemented - Medical treatments, so that I understood their relevance as well as how they would be implemented - The drugs I needed, so that I understood their effects, and how they should be administered - Routine issues of the ward (e.g. visiting hours, meals, rounds, etc.) - The medical examination and test results - The medical treatment results - Self-care procedures   The possibility to participate in the decision-making process regarding my medical care.  The possibility to participate in the decision-making process regarding my personal care.  The feeling that my knowledge of my illness/disease was taken into consideration.  The feeling that the doctors were interested in my uplifts.  The feeling that the doctors were interested in my concerns and hassles.  The feeling that the doctors were interested in my home situation.  The feeling that the doctors exhibited a sense of commitment.  The feeling that the nurses and assistant nurses were interested in my uplifts.  The feeling that the nurses and assistant nurses were interested in my concerns and hassles.  The feeling that the nurses and assistant nurses were interested in my home situation.  The feeling that the nurses and assistant nurses exhibited a sense of commitment.  The feeling that the doctors fully understood my situation.  The feeling that the doctors were personal in their contact with me.  The feeling that the doctors showed sympathy when I was suffering (e.g. from anxiety, pain, etc.).  The feeling that the nurses and assistant nurses fully understood my situation.  The feeling that the nurses and assistant nurses were personal in their contact with me.  The feeling that the nurses and assistant nurses showed sympathy when I was suffering.  The feeling that I have been treated with respect by the doctors.  A feeling of confidence that I received sincere answers to my questions from the doctors.  The feeling that I was treated in a positive manner by the doctors.  The feeling that I have been treated with respect by the nurses and assistant nurses.  A feeling of confidence that I received sincere answers to my questions from the nurses and assistant nurses.  The feeling that I was treated in a positive manner by the nurses and assistant nurses.  Sociocultural atmosphere  The experience that my integrity was protected when my medical records were handled.  The feeling that my family and friends were treated in a positive manner by the doctors.  The feeling that my family and friends were treated in a positive manner by the nurses and assistant nurses.  The possibility to meet with my family and friends at the times when it was most convenient for us.  The feeling that my desires and needs regarding wake-up time were not restricted by the ward.  The feeling that my desires and needs regarding bedtime were not restricted by the ward.  The feeling that my desires and needs regarding when to a shower were not restricted by the ward. | 1  4  5  5  1  1, 2  1  1  3  1, 2  1  1  3  1  3  2  1  3  2  1  3, 4  3  1  3, 4  3  1  1  1  1  1  1  1 |
| QPP Short Form (24) | | | | Larsson, 2002 (Sweden) | Patients aged ≥ 16 years who have received care for at least two days in the medical and surgical departments of two Swedish hospitals. |  |  | Item responses range from 1 (“Do not agree at all”) to 4 (“Completely agree”).  Identity-oriented approach  I received useful information on   - How examinations and treatments would take place - The results of examinations and treatments - Self-care; ‘how I should take care of myself’ - Which doctors were responsible for my medical care - Which nurses were responsible for my nursing care   I had good opportunity to participate in the decisions that applied to my care.  The doctors showed commitment; ‘cared about me’.  The nurses and assistant nurses showed commitment; ‘cared about me’.  The doctors seemed to understand how I experienced my situation.  The nurses and assistant nurses seemed to understand how I experienced my situation.  The doctors were respectful towards me.  The nurses and assistant nurses were respectful towards me.  Sociocultural atmosphere  My relatives and friends were treated well.  My care was determined by my own requests and needs rather than the staff’s procedures. | 4  5  3  3  1  1  1  1  1  1, 6 |
| QPP for Palliative Care (QPP-PC) (52) | | | | Sandsdalen, 2015 (Norway)* | Patients who have an advanced, life-threatening illness in a late palliative phase (malignant or non-malignant) and have received care for at least three days from inpatient hospices, hospice day-care centers, palliative units in nursing homes, or home-care districts that provide non-specialized palliative care services in Norway. | Patients assess healthcare staff (physicians, nurses, assistant nurses, priests, physiotherapists, occupational therapists or social workers). | | Item responses range from 1 (“Do not agree at all”) to 4 (“Fully agree”).  Medical-technical competence  I receive the best possible help for:   - Depression - Anxiety   Identity-oriented approach  I receive useful information on   - How care and treatments will take place - The effects and use of medicine - My illness and my symptoms - What I may expect in the near future (development of the illness and symptoms, my health and function) - How to take care of myself - Which doctor are responsible for my medical care - Which nurses are responsible for my nursing care   The personnel seem to give me honest answers to my questions:   - Doctors Nurses Other personnel   The personnel seem to understand how I experience my situation:   - Doctors - Nurses - Other personnel   The personnel are respectful towards me:   - Doctors - Nurses - Other personnel   I have good opportunity to participate in the decisions that apply to:   - Medical care - Nursing care - My individual plan for my care - Choosing where to receive my care   Sociocultural atmosphere  The personnel support and assist me in living the rest of my life in a meaningful way:   - Doctors - Nurses - Other personnel     The personnel support and assist me in tending to my spiritual and existential needs:   - Doctors - Nurses - Other personnel   My relatives:   - And friends are treated with respect - Receive the best possible help, support and care - May participate in decisions about my care, according to my preferences   My care is determined by my own requests and needs rather than staff procedures. | 2  4  4  1  1  5  6  1  1  1  1, 5  1, 6 |
| QPP-PC Short Form (24) | | | | Sandsdalen, 2020 (Norway) |  |  |  | Item responses range from 1 (“Do not agree at all”) to 4 (“Fully agree”).  Identity-oriented approach  I receive useful information on   - How care and treatments will take place - The effects and use of medicine - My illness and my symptoms - What I may expect in the near future (development of the illness and symptoms, my health and function) - How to take care of myself   The personnel give me honest answers to my questions.  The personnel understand how I experience my situation.  The personnel are respectful towards me.  I have good opportunity to participate in the decisions that apply to medical and nursing care.  Sociocultural atmosphere  The personnel support me in living my life in a meaningful way.  The personnel support me in tending to my spiritual and existential needs (life questions).  My care is determined by my own requests and needs rather than staff procedures. | 4  4  1  1  5  6  1  1, 6 |
| ***27) Quality Indicators for Palliative Care (Q-PAC) (31)*** | | | | | | | | | |
| Leemans, 2015 (Belgium) | | | | | Patients (who are living or have died within the past six weeks to six months) who are or have received support from palliative care services in Belgium: palliative home care teams, palliative care units, multidisciplinary mobile palliative support teams in hospitals and palliative reference nurses in care homes. | Patients or proxies, professional caregivers, bereaved family members, professional caregivers, and coordinators of the team assess nurses, physicians or psychologists of the palliative care team. | | Items are measured differently according to the type of question:  *[Patient items]*  Have your carers checked how you are feeling? (Three categories from “Yes, regularly” to “No”)  Did your carers give you freedom to plan your day? (Four categories from “Never” to Always” or “Not applicable”)  Can you co-decide about your care? (Three categories from “Never” to “Usually)  Do your carers take your personal wishes into account? (Four categories from “None of them do” to “All of them do”)  Are you getting enough information about…? (Three categories from “Less than necessary” to “More than enough”)   - Diagnosis - The course of the disease - With regard to end-of-life care   Do your carers explain things to you understandably? (Four categories from “Never” to Always”)  Do your carers give you conflicting information? (Four categories from “Never” to Always”) | 1  6  5  1  4  4  4 |
| ***28) Quality of End-of-life Care and Satisfaction with Treatment (QUEST)*** | | | | | | | | | |
| QUEST Scale: Patient Version (15) | | Sulmasy, 2002 (USA) | | | Internal medicine inpatients who are likely to die in teaching hospitals in the U.S (Maryland, New York). Diagnoses included malignancy, HIV, cardiopulmonary, or other. | | Patients assess all doctors and nursing staff with whom they had encounters, including nurse aides and technicians. | Quality items are rated using a five-point scale from “Never” to “Always.” Satisfaction items were rated from 1 (“Very dissatisfied”) to 5 (“Very satisfied”).  Over the past two days, how often have the doctors/nurses…  Spent enough time with you.  Seemed distracted by other things when you talk.  Been willing to take time to listen.  Treated you more as a disease than as a person.  Showed personal concern about you.  Ignored your feelings.  Responded quickly in time of need.  Over the past two days, how satisfied have you been with your doctors’/nurses’…  Bedside manner.  Way of talking to you.  Concern for you as an individual. | 3  1, 3  3  1  1  2  1  3  3  1 |
| QUEST Survey (15) | | Von Gruenigen, 2006 (USA) | | | Patients with recurrent gynecologic malignancies receiving chemotherapy in an oncology clinic office in the U.S. | | Patients assess physicians and nurses. |  |  |
| ***29) Quality of End-of-Life Communication (QOC)*** | | | | | | | | | |
| QOC (13) | | Engelberg, 2006 (USA) | | | Hospice patients from inpatient and outpatient hospice programs with survival prognosis of > two weeks.  Patients from ambulatory pulmonary clinics with COPD prescribed oxygen therapy for continuous home use. | | Patients assess doctors. | Item responses range from 0 (“The very worst I could imagine”) to 10 (“The very best I could imagine”) or “My doctor did not do this”/“Don’t know.”  When talking with Doctor (X) about important issues like becoming very ill, how good is he/she at:  Using words you understand?  Looking you in [the] eye?  Answering all questions about your illness [and treatment]?  Listening to what you have to say?  Caring about you as a person?  Giving [you his/her] full attention?  Talking [with you] about your feelings about getting sicker?  Talking [to you] about details if you got sicker?  Talking [to you] about how long you have to live?  Talking [to you] about what dying might be like?  Involving you in treatment discussions about your care?  Asking you about important things in life?  Asking about spiritual or religious beliefs?  *[Additional or different items in Panfilis, 2023]*  Including your loved ones in decisions about your illness and treatment?  Involving you in the decisions about the treatments that you want if you get too sick to speak for yourself?  Respecting the things in your life that are important to you?  Respecting your spiritual or religious beliefs?  How comfortable do you feel your doctor is [in] talking about dying? (0-10 numeric scale from “Not at all comfortable” to “Extremely comfortable”)  Overall, how would you rate this doctor’s communication with you? | 4  3  4  1, 3  1  1, 3  1, 2  4  4  4  5  1  1  1, 5  1, 5  1  1  4  3, 4 |
| Italian translation of QOC (19) | | De Panfilis, 2023 (Italy) | | | Patients enrolled at palliative care centers with inpatient, outpatient, and home-care facilities in Italy. | | Patients and their significant others assess doctors. |  |  |
| ***30) Quality of Life at the End of Life (QUAL-E)*** | | | | | | | | | |
| QUAL-E (24) | | Steinhauser, 2002 (USA) | | | Patients from a Veterans Administration and Duke University Medical Centers in the U.S (North Carolina) with stage IV cancer, CHF with ejection fraction of 20% or less, COPD, or dialysis-dependent ESRD. | | Patients assess doctors and other healthcare providers (nurses, social workers, chaplains, and hospice volunteers). | Item responses range from 0 (“Not at all”) to 5 (“Completely”).  Relationship with healthcare provider  I participate as much as I want in the decisions about my care.  Although I cannot control certain aspects of my illness, I have a sense of control about my treatment decisions.  Beyond my illness, my doctor has a sense of who I am as a person. | 5  6  1 |
| QUAL-E (26) | | Steinhauser, 2004 (USA) | | |  |  |  |  |  |
| QUAL-E Cancer measure (QUAL-EC) (17) | | Lo, 2010 (Canada) | | | Patients from outpatient oncology clinics in a Canadian hospital with advanced cancer (metastatic gastrointestinal, genitourinary, breast, lung or gynaecological cancer) and a prognosis of six months to two years. Patients with metastatic breast or prostate cancer were refractory to hormonal therapy. | |  |  |  |
| QUAL Cancer-Psychosocial (QUAL-EC-P) (14) | | Grünke, 2017 (Germany) | | | Patients with an incurable advanced tumor (Stage III or IV) who suffered from clinically meaningful psychological distress at an outpatient clinic for psycho-oncology or outpatient cancer counseling center at German university medical centers, the universities’ cancer care units or external cancer care facilities. | | Patients assess the healthcare team. |  |  |
| ***31) Schwartz Center Compassionate Care Scale (SCCCS) (12)*** | | | | | | | | | |
| Lown, 2015 (USA) | | | | | Patients recently hospitalized for at least three days in the U.S. | Patients assess healthcare providers, including doctors and nurses. | | Items responses range 1 (“Not at all successful”) to 10 (“Very successful”).  During your recent hospitalization, how successfully did your doctor or other healthcare provider:  Express sensitivity, caring and compassion for your situation?  Strive to understand your emotional needs?  Consider the effect of your illnesses on you and your family?  Listen attentively to you?  Convey information in a way that is understandable?  Gain your trust?  Always involve you in decisions about your treatment?  Treat you as a person not just as a disease?  Show respect for you and your family [and those important to you]?  Communicate test results in a timely and sensitive manner?  Spend enough time with you? | 2  2  1  1, 3  4  3  5  1  1  4  3 |
| Lown, 2017 (Ireland) | | | | | Recently hospitalized and non-hospitalized patients in Ireland. |  |  |  |  |
| Rodriguez, 2019 (USA) | | | | | Patients with cancer, neurologic disorders (Amyotrophic Lateral Sclerosis, multiple sclerosis, Parkinson’s disease), heart disorders (coronary artery disease, hypertension, congestive heart failure, cardiomyopathy), and lung and respiratory disorders (pulmonary fibrosis, asthma, COPD, cystic fibrosis, emphysema, pulmonary hypertension), or a mental illness. Patients had to have been hospitalized within the last 18 months in the U.S. |  |  |  |  |
| Zeray, 2021 (Ethiopia) | | | | | Oncology patients receiving follow-up care or admitted for a minimum of two days at a specialized hospital in Ethiopia. |  |  |  |  |
| ***32) Sinclair Compassion Questionnaire (SCQ) (15)*** | | | | | | | | | |
| Sinclair, 2021 (Canada) | | | | | Patients or care residents with a life-limiting illness (e.g., cancer, chronic obstructive pulmonary disease, coronary heart disease, dementia) in acute care, hospice, long-term care and home care settings in Canada. | Patients assess healthcare providers. | | Items responses range from 1 (“Strongly disagree”) to 5 (“Strongly agree”):  Feel cared for.  Genuine concern.  Communicated sensitive.  Attentive.  Provided comfort.  Very supportive.  Provided care.  Spoke with kindness.  Saw as person.  Behaved in caring way.  Really understood needs.  Good relationship.  See my perspective.  Warm presence.  Sincere. | 3  1  2  3  2  3  3  3  1  3  1  3  1  3  3 |
