# Supplementary material for: Measuring the quality of patient-provider relationships in serious illness: A scoping review
Source: Palliat Med. 2025 Feb 6;39(3):332–45. doi: 10.1177/02692163251315304 (PMC11877987; doi:10.1177/02692163251315304)
Supplement: sj-docx-4-pmj-10.1177_02692163251315304 – Supplemental material for Measuring the quality of patient-provider relationships in serious illness: A scoping review [file sj-docx-4-pmj-10.1177_02692163251315304.docx]

| **Table 2B. Measures for caregivers, relatives, or other respondents** | | | | | |
| --- | --- | --- | --- | --- | --- |
| **Measure name (number of items)** | | | | | |
| **Author, year (location)** | | **Patient population and study setting** | **Respondent and provider** | **Relational item(s) and measure format** | **Element of relationship quality** |
| ***1) Bereaved Family Survey in Community Nursing Homes (BFS-CNH) (10)*** | | | | | |
| Kinder, 2022 (USA) | | Deceased veterans who had received hospice care in community nursing homes that offer hospice services. | Veterans’ next-of-kin assess healthcare providers. | Likert-type or dichotomous responses and two open-ended questions. Four-point scale from 0 (“Never”) to 3 (“Always”). Global rating item measured by an 11-point scale of 0 (“Worst Care Possible”) to 10 (“Best Care Possible”).  Care and Communication  Providers listened to concerns.  Providers gave treatment patient wanted.  Providers were kind, caring, and respectful.  Providers kept family members informed about condition and treatment.  Emotional and Spiritual Support  Providers gave enough spiritual support.  Providers gave enough emotional support before death. | 1  1  1, 3  1, 4  1  2 |
| ***2) Canadian Health Care Evaluation Project (CANHELP) Questionnaire*** | | | | | |
| CANHELP (caregiver version: 40) | Heyland, 2010 (Canada) | Patients from inpatient and outpatient facilities and home care programs in Canada who are (a) > 55 years old with COPD, CHF, cirrhosis and/or metastatic cancer or (b) ≥ 80 years old and admitted to a hospital or (c) enrolled in a home care program using long-term oxygen therapy and have COPD, CHF, or metastatic cancer. | Patients and caregivers assess doctors, nurses, and other healthcare professionals. | Degree of satisfaction is rated from 1 (“Not at all satisfied”) to 5 (“Completely satisfied”).  How satisfied are you (with respect to the last month of care)…  the way you were treated by the doctors and nurses looking after your relative?  Relationship with the doctors  that the doctor(s) took a personal interest in your relative?  that the doctor(s) were available when you or your relative needed them?  with the level of trust and confidence you had in the doctor(s) who looked after your relative?  Characteristics of the doctors and nurses  with the level of trust and confidence you had in the nurses who looked after your relative?  that the doctors and nurses looking after your relative were compassionate and supportive of him or her?  that the doctors and nurses looking after your relative were compassionate and supportive of you?  that your relative was treated by those doctors and nurses in a manner that preserved his or her sense of dignity?  Illness management  that emotional problems your relative had were adequately controlled?  that the care and treatment your relative received was consistent with his or her wishes?  Communication and decision making  that the doctor(s) explained things relating to your relative’s illness in a straight-forward, honest manner?  that the doctor(s) explained things relating to your relative’s illness in a way you could understand?  that you received consistent information about your relative’s condition from all the doctors and nurses looking after him or her?  that you received updates about your relative’s condition, treatments, test results, etc. in a timely manner?  that the doctor(s) listened to what you had to say?  with discussions with the doctor(s) about where your relative would be cared for if he or she were to get worse?  Your involvement  with discussions with the doctor(s) about the use of life sustaining technologies?  with your role during the past month in decision-making regarding your relative’s medical care? | 1  1  1, 3  3  3  3  1  1  2  1  4  4  4  4  1  4  4  1, 5 |
| CANHELP-Lite (caregiver version: 23) | Heyland, 2013 (Canada) |  |  | Degree of satisfaction is rated from 1 (“Not at all satisfied”) to 5 (“Completely satisfied”).  In general, how satisfied are you with the way you were treated by the doctors, nurses, and other healthcare professionals looking after your relative?  How satisfied are you (with respect to the last month of care)…  Relationship with the doctors  that the doctor(s) took a personal interest in your relative?  that the doctor(s) were available when you or relative needed them?  with the level of trust and confidence you had in the doctor(s) who looked after your relative?  Characteristics of the doctors and nurses  that the doctors, nurses, and other healthcare professionals who looked after your relative were compassionate and supportive of him or her?  that the doctors, nurses, and other healthcare professionals who looked after your relative were compassionate and supportive of you?  Illness management  that you were treated by those doctors, nurses, and other healthcare professionals in a manner that preserved your sense of dignity?  that the emotional problems your relative had were adequately assessed and controlled?  that the care and treatment your relative received was consistent with his or her wishes?  Communication and decision making  that the doctor(s) explained things relating to your relative’s illness in a straight-forward, honest manner?  that you received consistent information about your relative’s condition from all the doctors and nurses looking after him or her?  that the doctor(s) listened to what you had to say?  with discussions with the doctor(s) about where your relative would be cared for if he or she were to get worse?  Your involvement  with discussions with the doctor(s) about the use of life sustaining technologies?  with your role in decision-making regarding your relative’s medical care? | 1  1  1, 3  3  3  1  1  2  1  4  4  1  4  4  1, 5 |
| ***3) Care Evaluation Scale (CES)*** | | | | | |
| CES (28) | Morita, 2004 (Japan) | Cancer patients who died in palliative care units in Japan. | Bereaved family members assess physicians and nurses. | Necessity for improvement for each item is rated on a six-point Likert scale from 1 (“Improvement is not necessary”) to 6 (“Highly necessary”) (Morita, 2004). Five-point Likert scale from 1 (“worst”) to 5 (“best”) or eleven-point numerical rating scale from 0 (“Worst”) to 10 (“Best”) (Benitez-Rosario, 2015).  Physical care by nurse  Nurses helped the patient to enjoy daily life (recreation, music, hobbies).  Psycho-existential care  Consideration was paid to relieving the patient’s concerns and worries.  The staff took appropriate measures when the patient became depressed.  The staff tried so that the patient’s hope could be accomplished.  Help with decision-making  The doctors gave sufficient explanation to the patient about their present condition and the details of medical treatment.  The doctors gave sufficient explanation to the patient about the expected outcome.  Consideration was given so that the patient could participate in the selection of treatment.  The doctors gave sufficient explanation to the family about the patient's condition and the details of medical treatment.  The doctors gave sufficient explanation to the family about the expected outcome.  The family's wishes were respected in the selection of treatment.  Family burden  Consideration was given to the health of the family.  Consideration was given so that the family could have their own time and continue to work.  Availability  Admission (use) was in accordance with the wishes of the patient and family. | 1  1  2  2  4  4  5  1, 4  1, 4  1  1  1  1 |
| Spanish CES (26) | Benitez-Rosario, 2015 (Spain) | Patients with advanced cancer in an inpatient palliative care unit in Spain. | Relatives assess staff members including doctors and nurses. |  |  |
| ***4) Caregiver Evaluation of the Quality of End-Of-Life Care (CEQUEL) Scale (13)*** | | | | | |
| Higgins, 2013 (USA) | | Terminally ill cancer patients aged ≥ 20 years in seven outpatient sites in the U.S. | Caregivers assess any doctor or other medical staff. | Two options presented as checkboxes for each question:  Was there ever a problem understanding what any doctor was saying to you about what to expect from treatment? (“Yes/No”)  Was there any medical procedure or treatment that happened to (him/her) that was inconsistent with (his/her) previously stated wishes? (“Yes/No”)  Did you feel that the doctors you talked to listened to your concerns about [Patient’s] medical treatment? (“Yes/No”)  How often were you or other family members kept informed about [Patient’s] condition? (“Sometimes/Never” or “Usually/Always”) | 4  1  1  1, 4 |
| ***5) CaregiverVoice Survey (62)*** | | | | | |
| Seow, 2017 (Canada)* | | Deceased patients who had received palliative care services in either the home or residential hospice. Main diagnoses of patients included cancer, kidney or liver disease, heart disease, Alzheimer’s or other neurological diseases, COPD/asthma, stroke. | Bereaved caregivers | Item responses presented as checkboxes. Item choices vary depending on the question type.  *Note: Measure also comprises FAMCARE-2 items (see FAMCARE-2)*  Overall, do you feel that you and your family got as much help and support from homecare services as you needed? (“Yes/No”).  During the last 3 months of his/her life, while he/she was receiving homecare services, what is your assessment of the overall level of support given in the following areas? (“Excellent,” “Very good,” “Fair,” “Poor,” “Does not apply,” “Don’t know”)   - Spiritual support - Emotional support - Respect and dignity   During the last 3 months of his/her life, while he/she was being cared for by the clinician you selected above, what is your assessment of the overall level of support given in the following areas?   - Spiritual support - Emotional support - Respect and dignity   During the last 3 months of his/her life, when he/she was being cared for by the cancer center you named, what is your assessment of the overall level of support given in the following areas?   - Spiritual support - Emotional support - Respect and dignity     During the last 3 months of his/her life, while he/she was in a Long Term Care home, what is your assessment of the overall level of support given in the following areas?   - Spiritual support - Emotional support - Respect and dignity   During his/her last hospital admission, what is your assessment of the overall level of support given in the following areas?   - Spiritual support - Emotional support - Respect and dignity   During the last 3 months of his/her life, while he/she was in hospice, what is your assessment of the overall level of support given in the following areas?   - Spiritual support - Emotional support - Respect and dignity   Thinking about the setting you selected above, indicate your opinion about the help he/she received in the last week of life to each of the following:   - There was enough help available to meet his/her personal care needs - There was enough support to stay where he/she wanted to be   During the last week of life for the setting you specified, what is your assessment of the level of support given in the following areas?   - Spiritual support - Emotional support - Respect and dignity   Were you or his/her family given enough help and support by the healthcare providers at the actual time of his/her death? (“Yes definitely/to some extent,” “No not at all,” “Don’t know”)  Looking back over the last 3 months of his/her life, was he/she involved in decisions about his/her care as much as he/she would have wanted? (“He/she was involved as much as he/she wanted to be,” “would have liked to be more/less involved,” “wasn’t able to be involved,” “Don’t know”)  Looking back over the last 3 months of his/her life, were you involved in decisions about his/her care as much as you would have wanted? (“I was involved as much as I wanted to be,” “would have liked to be more/less involved,” “Don’t know”)  Were any decisions made about his/her care that he/she would not have wanted? (“No,” “Yes,” “Don’t know”)  Was he/she given the opportunity to discuss advance care planning with his/her healthcare providers? (“Yes definitely/to some extent,” “No,” “Don’t know”)  Did your healthcare providers help you, the caregiver, understand what to expect/how to prepare for his/her death? (“Yes definitely/to some extent,” “No,” “Don’t know”) | 1  1  2  1  1  2  1  1  2  1    1  2  1    1  2  1  1  2  1  1  1  2  1  1  5  1, 5  1  5  1, 4 |
| ***6) Consumer Assessment of Healthcare Providers and Systems (CAHPS®)*** | | | | | |
| CAHPS Nursing Home Survey (21) | Frentzel, 2012 (USA) | Nursing home residents in the U.S (Texas). | Family members, friends, guardians, medical powers of attorney, and attorneys assess nurses and aides. | Item responses range from “Never” to “Always” or “Yes”/“No.”  How often did you see that the nurses/aides treat your family member with courtesy and respect?  How often did you see that the nurses/aides treat your family member with kindness?  How often did you feel that the nurses/aides really cared about your family member?  Did you ever see any nurses or aides be rude to your family member or any other resident?  How often did you get this information [about your family member from a nurse/aide] as soon as you wanted?  How often did the nurses/aides explain things in a way that was easy for you to understand?  How often were you involved as much as you wanted to be in the decisions about your family member’s care?  Did the nurses/aides ever try to discourage you from asking questions about your family member? | 1  3  3  1, 3  4  4  1, 5  1, 4 |
| CAHPS Hospice Survey (47) | Price, 2018 (USA) | Patients who died ≥ two days after admission to hospices in the U.S. | Primary caregivers assess the hospice team, including all the nurses, doctors, social workers, chaplains, etc. | Item responses range from “Never” to “Always” or “Yes”/“No” or from 0 (“Worst hospice care possible”) to 10 (“Best hospice care possible”).  How often did you get the help you needed from the hospice team during evenings, weekends, or holidays?  How often did the hospice team keep you informed about when they would arrive to care for your family member?  When you or your family member asked for help from the hospice team, how often did you get help as soon as you needed it?  How often did the hospice team explain things in a way that was easy to understand?  How often did the hospice team keep you informed about your family member’s condition?  How often did anyone from the hospice team give you confusing or contradictory information about your family member’s condition or care?  How often did the hospice team treat your family member with dignity and respect?  How often did you feel that the hospice team really cared about your family member?  How often did the hospice team listen carefully to you when you talked with them about problems with your family member’s hospice care?  How often did your family member get the help he or she needed from the hospice team for feelings of anxiety or sadness?  Did the hospice team give you as much information as you wanted about what to expect while your family member was dying?  How often was the information you were given about your family member by the nursing home staff different from the information you were given by the hospice team?  How often did the hospice team listen carefully to you? | 1  1, 4  1, 2  4  1, 4  4  1  3  1  2  4  4  1 |
| ***7) Critical Care Family Satisfaction Survey (CCFSS) (20)*** | | | | | |
| Wasser, 2001 (USA) | | Patients in a critical care unit – Medical Intensive Care, Shock Trauma, Acute Coronary Care, Central Nervous System, Surgical Intensive Care, and Special Care units of a hospital in the U.S (Pennsylvania). | Family members assess staff members including doctors and nurses. | Five-point Likert scale from 1 (“Very dissatisfied”) to 5 (“Very satisfied”).  Describe how satisfied you were with the care that you and your family member received while in the critical care unit:  Honesty of the staff about my family member’s condition.  Availability of the doctor to speak with me on a regular basis.  Ability to share in the care of my family member.  Clear explanation of tests, procedures, and treatments.  Support and encouragement given to me during my family member’s stay in the critical care unit.  Clear answers to my questions.  Sharing in decisions regarding my family member’s care on a regular basis.  Nurses’ availability to speak with me every day about my family member’s care.  Sensitivity of the doctor(s) to my family member’s needs.  Sharing in discussions regarding my family member’s recovery. | 4  1, 4  1, 5  4  1, 2  4  1, 5  1, 4  2  1, 5 |
| ***8) Direct Observation Checklist (DOC) to Measure Respect and Dignity in the ICU (10)*** | | | | | |
| Carrese, 2017 (USA) | | Patients in seven ICUs (medical, surgical, specialty-specific) within the Johns Hopkins Health System in the U.S. | Trained observers assess nurses, doctors, therapy specialists, technicians/other support staff, nurse practitioners/physician assistants, clergy/social workers. | Items were assigned a score of 1 for a response “Yes” and 0 for “No” or “N/A.” One item (description of general demeanor) is assessed as “Positive,” “Negative,” or “Neutral.” Global rating item responses range from “Not at all” to “To a great extent.”  Did the clinician greet the patient *(and visitor, if present)* when entering the patient’s room?  Was an effort made to explain an activity to the patient *(and visitor, if present)*?  Did the clinician make an attempt to be at the patient’s eye level when not engaged in activities that require standing?  Was the clinician responsive to the needs or requests of the patient *(and visitor, if present)*?  How would you describe the general demeanor of the clinician?    Would you describe the clinician’s demeanor as pleasant?  Would you describe the clinician’s demeanor as compassionate?  Would you describe the clinician’s demeanor as supportive?  Would you describe the clinician’s demeanor as distant?  Global rating: To what extent did this clinician treat the patient with respect and dignity? | 1, 3  1, 4  3  1  3  3  3  3  3  1 |
| ***9) euroQ2 Questionnaire (34)*** | | | | | |
| Jensen, 2015 (Denmark and Netherlands) | | Patients admitted to ICUs in Denmark and the Netherlands for ≥ 48 hours. | Family members (closest persons to patients including partners and friends) assess doctors, nurses, and other healthcare professionals and ICU staff. | Items are rated from “Excellent” to “Poor” with “Don’t know” and “N/A” options or numerically from 0 (“Worst care possible”) to 10 (“Best care possible”).  Concern and caring by ICU staff  The courtesy, respect, and compassion your family member was given.  Atmosphere of the ICU  How well the involved ICU staff made you feel that your presence was appreciated.  Consideration of your needs  How well the involved ICU staff showed an interest in your needs.  Emotional support  How well the involved ICU staff provided emotional support.  Ease of getting information  Willingness of ICU staff to answer your questions.  Understanding of information  How well ICU staff provided you with explanations that you understood.  Honesty of information  Perceived honesty of information provided to you about your family member’s condition.  Completeness of information  How well ICU staff informed you about what was happening with your family member.  How well ICU staff informed you about why things were being done to your family member.  Consistency of information  The consistency of information provided to you about your family member’s condition (Did you get a similar story from the doctor, nurse, etc.).  Overall quality of information  The overall quality of information provided to you by doctors.  The overall quality of information provided to you by nurses.  Inclusion in the decision-making processes  How well the staff involved you in major decision-making processes.  Support during the decision-making processes  How well ICU staff supported you when major decisions were made.  Do you think that your family member got the emotional support he/she needed?  Do you think that your family member got the spiritual support he/she needed?  Was the end-of-life care according to the wishes of your family member? | 1, 3  1  1  2  1, 4  1, 4  4  1, 4  1, 4  4  4  4  1, 5  1  2  1  1 |
| Jensen, 2017 (Denmark and Netherlands)* | |  |  |  |  |
| ***10) Family Assessment of Treatment at End of Life (FATE)*** | | | | | |
| FATE (32) | Casarett, 2008 (USA) | Patients who received inpatient or outpatient care from U.S Veterans Affairs (VA) medical centers in their last month of life. | Family members (next of kin, primary contact named in electronic medical record, and Durable Power of Attorney for Health Care) assess healthcare providers. | Item responses are scored as 1 (“Always”) or 0 (“Usually/”Sometimes”/“Never”) or “Yes”/“No responses.  *Items are only included in FATE unless specified otherwise.*  Well-being and dignity  Inpatient providers handled the patient gently.  Inpatient providers supervised the patient closely enough.  Inpatient providers offered comfortable accommodations for respondents.  Information and communication  Providers gave contradictory information.  *[FATE-S and FATE]* Providers spoke in an understandable way.  *[FATE-S and FATE]* Providers listened to concerns.  Providers were available to talk to the patient/family.  *[FATE-S and FATE]* Providers kept patient/family informed about the patient’s condition and treatment.  Respect for treatment preferences  Patient received desired medications or treatment.  Patient received unwanted medication or treatment /  *[FATE-S]* Providers gave treatment the patient did not want.  Emotional and spiritual support  *[FATE-S and FATE]* Providers were kind, caring, and respectful.  *[FATE-S and FATE]* Providers gave adequate spiritual support to patient/family.  *[FATE-S and FATE]* Providers gave adequate emotional support to patient/family prior to death.  Care around the time of death  The family had enough warning one month prior to patient’s death.  VA services  The patient/family received enough care at home. | 3  3  1  4  4  1  1  1, 4  1  1  1, 3  1  1, 2  1, 4  1 |
| Short Form (FATE-S) (14) | Casarett, 2010 (USA) | Patients who died in U.S Veterans Administration medical centers. |  |  |  |
| ***11) Family Perceptions of Physician-Family Caregiver Communication (FPPFC) (7)*** | | | | | |
| Koniewski, 2022 (Belgium, Finland, Italy, the Netherlands and Poland)* | | Nursing home residents in Belgium, Finland, Italy, the Netherlands, and Poland who had dementia, died due to cancer, and/or received palliative care and died in the last three months. | Relatives of nursing home residents assess doctors. | Response items are rated from 1 (“Strongly disagree”) to 4 (“Strongly agree”).  The doctor always kept you or other family members informed about resident’s condition.  Your relative’s doctor always helped you or other family members to understand what he or she was saying to you about what to expect while your relative was dying.  The doctor always spoke to you, other family members or your relative about your relative’s wishes for medical treatment at the end of life.  The doctor always listened to what you, other family members, or your relative had to say about his/her medical treatment and end-of-life care.  The doctor always understood what you, other family members, and your relative were going through. | 1, 4  1, 4  1  1,3  1 |
| ***12) Family Satisfaction in the Intensive Care Unit (FS-ICU) Survey (24)*** | | | | | |
| Wall, 2007 (Canada and USA) | | Canadian cohort: Critically ill patients in ICUs in university-affiliated tertiary hospitals.  U.S. cohort: Patients in ICUs in university-affiliated tertiary hospitals. for ≥6 hours. | Family members assess  ICU staff, including doctors, nurses, social workers. | Items are rated on a five-point scale from 1 (“Excellent”) to 5 (“Poor”) and a “N/A” option.  How satisfied are you with…  How did we treat your family member (the patient)?  The courtesy, respect, and compassion your family member was given.  The ICU staff  How well the nurses cared for your family member.  How often nurses communicated to you about your family member’s condition.  How well doctors cared for your family member.  Information needs  How often doctors communicated to you about your family member’s condition.  Willingness of ICU staff to answer your questions.  The honesty of information provided to you about your family member’s condition.  How well ICU staff informed you what was happening to your family member and why things were being done.  The consistency of information provided to you about your family member’s condition.  Did you feel included in the decision making process?  Did you feel supported during the decision making process?  Did you feel you had control over the care of your family member?  When making decisions, did you have adequate time to have your concerns addressed and questions answered? | 1, 3  1  1, 4  1  1, 4  1, 4  4  4  4  1, 5  1  1  1, 4 |
| Wright, 2015 (UK) | | Patients who spent ≥24 hours in general ICUs in England, Wales, and Northern England. |  |  |  |
| ***13) Family Satisfaction with End-of-Life Care (FAMCARE) Scale*** | | | | | |
| FAMCARE (20) | Kristjanson, 1993 (Canada) | Patients with advanced cancer in an outpatient oncology department and a home healthcare service. | Family members/informal caregivers assess health professionals, including doctors and nurses. | Item responses range from 1 (“Very satisfied”) to 5 (“Very dissatisfied”). In Alnjadat (2014), item choices are reversed from 1 (“Very dissatisfied”) to 5 (“Very satisfied”).  How satisfied are you with:  Information provided about prognosis.  Information given about side effects.  Family conferences held to discuss the patient’s illness.  Doctor’s attention to patient’s description of symptoms.  Availability of doctors to the family.  Availability of nurses to the family.  The way the family is included in treatment and care decisions.  Information given about how to manage the patient’s pain.  Information given about the patient’s tests.  Availability of doctor to the patient. | 4  4  4  1, 4  1  1  1, 5  4  4  3 |
|  | Ringdal, 2003 (Norway) | Patients with advanced cancer and a predicted survival of two to nine months at a palliative medicine unit in a Norwegian university hospital. |  |  |  |
|  | Rodriguez, 2009 (USA) | Patients in a geriatric palliative care unit and traditional nursing home care units at a VA medical center in Northeastern U.S. |  |  |  |
|  | Can, 2011 (Turkey) | Patients with cancer at a Turkish university institute of oncology. |  |  |  |
|  | Alnjadat, 2014 (Malaysia) | Cancer patients receiving outpatient oncology care at oncology clinic in Malaysian university hospital. |  |  |  |
|  | Teresi, 2014 (USA) | Hospitalized patients with advanced cancer in Eastern and Midwestern U.S hospitals. |  |  |  |
|  | Chattat, 2016 (Italy) | Cancer patients receiving cancer care, curative and/or palliative, in an oncology ward at Italian outpatient clinics and community-based hospice. |  |  |  |
| FAMCARE-2 (17) | Aoun, 2010 (Australia) | Patients with cancer, end-stage organ failure (cardiac, respiratory, renal and hepatic) or neurodegenerative diseases receiving inpatient or community palliative care services in Australia. | Family caregivers assess the palliative care team. | Item responses range from 1 (“Very satisfied”) to 5 (“Very dissatisfied”).  How satisfied are you with:  Management of physical symptoms and comfort  Palliative care team’s attention to the patient’s description of symptoms.  The way in which the patient’s physical needs for comfort are met.  The doctor’s attention to the patient’s symptoms.  Provision of information  The way in which the patient’s condition and likely progress have been explained by the palliative care team.  Information given about the side effects of treatment.  Meetings with the palliative care team to discuss the patient’s condition and plan of care.  Information given about how to manage the patient’s symptoms (e.g. pain, constipation).  Family support  Availability of the palliative care team to the family.  Emotional support provided to family members by the palliative care team.  The way the family is included in treatment and care decisions.  Patient psychological care  The way in which the palliative care team respects the patient’s dignity.  The palliative care team’s response to changes in the patient’s care needs.  Emotional support provided to the patient by the palliative care team. | 1, 4  1  1, 4  4  4  4  4  1  1, 2  1, 5  1  1  2 |
|  | D’Angelo, 2017 (Italy) | Patients who have been enrolled for at least three days in a palliative care unit in Italy. |  | Five-point Likert-type scale ranging from 1 (“Strongly disagree”) to 5 (“Strongly agree”).  The way in which the patient’s condition and likely progress have been explained by the palliative care team.  Information given about the side effects of treatment.  The way in which the palliative care team respects the patient’s dignity.  Meetings with the palliative care team to discuss the patient’s condition and plan of care.  Palliative care team’s attention to the patient’s description of symptoms.  The way in which the patient’s physical needs for comfort are met.  Availability of the palliative care team to the family.  Emotional support provided to family members by the palliative care team.  The doctor’s attention to the patient’s symptoms.  The way the family is included in treatment and care decisions.  Information given about how to manage the patient’s symptoms.  The palliative care team’s response to changes in the patient’s care needs.  Emotional support provided to the patient by the palliative care team. | 4  4  1  4  1, 4  1  1  1, 2  1, 4  1, 5  4  1  2 |
| FAMCARE-6 (6) | Carter, 2010 (Australia) | Ambulatory oncology patients in an Oncology–Haematology outpatient clinic in Australia. | Caregivers assess health professionals. | Item responses range from 1 (“Very satisfied”) to 5 (“Very dissatisfied”).  How satisfied are you with:  Information given about side effects.  Availability of doctors to the family.  Availability of the doctor to the patient. | 4  1  3 |
| FAMCARE-5 (5) | Ornstein, 2015 (USA) | Hospitalized patients with advanced cancer in U.S hospitals. | Family members assess doctors. | Item responses are “Very satisfied”, “Satisfied” or “Not satisfied.”  How satisfied are you with the following:  Doctor’s attention to patient’s description of symptoms.  Information given about the patient’s tests.  Availability of the doctor to the patient.  *[Additional items in FAMCARE-10]*  Availability of doctors to the family.  The way the family is included in treatment and care decisions.  Information given about how to manage the patient’s pain. | 1, 4  4  3  1  1, 5  4 |
| FAMCARE-10 (10) |  |  |  |  |  |
| Spanish version of Short-form FAMCARE (10) | Teresi, 2019 (USA) | Patients with Alzheimer’s disease and related dementias (ADRD) in the U.S (New York). | Caregivers assess doctors. | Item responses are coded as 2 (“Very satisfied”), 1 (“Satisfied”) and 0 (indecision or dissatisfaction).  How satisfied are you with:  The doctor’s attention to your description of your (care recipient’s) symptoms?  Availability of doctors to the family?  The way the family is included in treatment and care decisions?  Information given about how to manage your (care recipient’s) pain?  Information given about your (care recipient’s) tests?  Availability of doctor to your (care recipient)? | 1, 4  1  1, 5  4  4  3 |
| ***14) Information Sharing and Communication (ISC) Scale (7)*** | | | | | |
| Brazil, 2013 (Canada) | | Patients who died in tertiary acute care teaching hospitals in Canada. | Patients’ next-of-kin assess the healthcare team. | Items are rated on a five-point Likert scale from 1 (“Not at all satisfied”) to 5 (“Completely satisfied”).  The healthcare team’s knowledge of the patient’s wishes for end of life care.  The quality of the discussions with the healthcare team about the use of life-sustaining technologies. | 1  4 |
| ***15) Palliative Care Transition Measure for Caregivers (PCTM-C) (15)*** | | | | | |
| D’Angelo, 2018 (Italy) | | Patients that had transferred from an acute care to palliative care centers in Italy. | Family caregivers assess health professionals. | Item responses range from 1 (“Completely disagree”) to 4 (“Completely agree”).  Before my relative left the hospital, health professionals took my preferences and those of my relative into account in deciding the place of referral to palliative care.  Before my relative left the hospital, health professionals took my preferences and those of my relative into account in organizing the referral to palliative care.  When my relative left the hospital, I was confident that his/her symptoms would be managed as well as possible.  When my relative left the hospital, I was confident that we would receive all the help needed.  Health professionals supported us throughout the referral to palliative care. | 1  1  1  1  1 |
| ***16) Quality Indicators for Palliative Care (Q-PAC) (31)*** | | | | | |
| Leemans, 2015 (Belgium) | | Patients (who are living or have died within the past six weeks to six months) who are or have received support from palliative care services in Belgium: palliative home care teams, palliative care units, multidisciplinary mobile palliative support teams in hospitals and palliative reference nurses in care homes. | Patients or proxies, professional caregivers, bereaved family members, professional caregivers, and coordinators of the team assess nurses, physicians or psychologists of the palliative care team. | Items are measured differently according to the type of question:  Did you receive information about your relative’s condition? (Three categories from “Less than necessary” to “More than enough”)  Did you get information about the pros and cons of different treatments? (Three categories from “Less than necessary” to “More than enough”)  Did you get information about the impending death? (Three categories from “Less than necessary” to “More than enough”)  Did the carers take each of these questions about a treatment decision seriously? (Two categories “Yes or “No”)  Did you feel that the carers gave you all the help and support you needed to care for your relative? (Five categories from “Yes, I received all the help I needed” to “I did not need any help”)  Did the carers ask how you were feeling? (Three categories from “Yes, regularly” to “No”) | 1, 4  1, 4  1, 4  1, 4  1  1 |
| ***17) Quality of Dying in Long-Term Care (QOD-LTC and QOD-LTC-C) (36)*** | | | | | |
| Munn, 2007 (USA) | | Cognitively intact and impaired residents of nursing homes and residential care or assisted living facilities in the U.S (Florida, Maryland, North Carolina, New Jersey). | Primary family and staff caregivers assess physicians, nurses, and aides. | Item responses range from 1 (“Not at all”) to 5 (“Completely”) or “Don’t know.”  There was a nurse or aide with whom [Resident] felt comfortable.  [Resident’s] physician knew [him/her] as a whole person.  [Resident] had a physician whom [he/she] trusted.  [Resident] had as much information as [he/she] wanted about [his/her] illness.  Although [he/she] could not control certain aspects of [his/her] illness, [Resident] had a sense of control about [his/her] treatment decisions.  [Resident] participated as much as [he/she] wanted in the decisions about [his/her] care. | 3  1  3  4  6  5 |
| ***18) Quality of Life at the End of Life (QUAL-E)*** | | | | | |
| QUAL-E Fam (17) | Steinhauser, 2014 (USA) | Patients from a Veterans Administration and Duke University Medical Centers in the U.S (North Carolina) with advanced cancer, CHF, COPD, or other life-limiting illness. | Family members assess the healthcare team. | How often does the healthcare team…  involve you in making decisions about patient’s care?  keep you informed about patient’s condition?  respond to your concerns about [Patient]? | 1, 5  1, 4  1 |
| ***19) Satisfaction Scale for Family Members Receiving Inpatient Palliative Care (Sat-Fam-IPC) (34)*** | | | | | |
| Morita, 2002 (Japan) | | Cancer patients who had died in palliative care units in Japan. | Informal carers assess physicians, nursing staff, and ward staff. | Item responses range from 0 (“Very dissatisfied”) to 6 (“Very satisfied”).  Nursing care  The nurses and assistant nurses provided sufficient help with daily care for the patient.  The patient’s and the family’s wishes were reflected in the care of the patient.  The patient’s dignity was protected during the care.  Attention was continuously paid to the patient’s suffering.  The staff was available for consultations about concerns and anxieties when death was close.  The ward staff provided enough time in the care of the patient.  The demeanor of the ward staff was always warm.  Complaints and requests were handled smoothly.  Availability  Admission was in accordance with the wishes of the patient.  Admission was in accordance with the wishes of the family.  Information  The physician explained the treatment to alleviate the patient’s discomfort.  The physician’s explanations were easy to understand.  Symptom palliation  The staff responded not only to physical distress but also emotional distress such as anxieties. | 1  1  1  2  1  3  3  1  1  1  4  4  1, 2 |
| ***20) Satisfaction with care End of Life in Dementia (SWC-EOLD) (10)*** | | | | | |
| Volicer, 2001 (USA) | | Patients from chapters of the Alzheimer’s Association, Veterans Administration Geriatric Research Educations Clinical Centers and the National Institute on Aging Alzheimer’s Disease Centers in the U.S who had dementia and died within one year. | Caregivers assess the healthcare team, including doctors and nurses. | Four-point Likert scale ranging from 1 (“Strongly disagree”) to (“Strongly agree”).  All measures were taken to keep my care recipient comfortable.  The healthcare team was sensitive to my needs and feelings.  I feel that all medication issues were clearly explained to me.  I felt fully involved in all decision making. | 1  1, 2  1, 4  1, 5 |
| Kiely, 2006 (USA) | | People aged > 65 years with dementia/ dying with advanced dementia in nursing homes (for ≥ 30 days in Kiely, 2006) in Massachusetts, USA. | Healthcare proxies assess the healthcare team, including doctors and nurses. |  |  |
| Kiely, 2011 (USA) | |  |  |  |  |
| ***21) Toolkit After-Death Bereaved Family Member Interview (121)*** | | | | | |
| Teno, 2001 (USA) | | Patients who died in an outpatient hospice service, nursing homes, or an academic medical center in the U.S (New England region). | Bereaved family members doctors, nurses, and other professional staff. | Items are measured differently according to the type of question:  In [Patient’s] last week/While under care of hospice/During the last month of life…  Was there ever a problem understanding what any doctor was saying to you about what to expect from treatment? (“Yes”/“No”)  Did you feel that the doctors you talked to listened to your concerns about [Patient’s] medical treatment? (“Yes”/“No”/“Had no concerns”)  How much information did the doctors provide you about [Patient’s] medical condition - would you say less information than was needed, just the right amount, or more than was needed?  How often did any doctor give confusing or contradictory information about [Patient’s] medical treatment always, usually, sometimes, or never?  To the best of your knowledge, did [Patient’s] doctor or the medical staff who cared for (him/her) while under care of hospice speak to (him/her) or you about (his/her) wishes about medical treatment? (“Yes”/“No”)  Did (his/her) doctor or the medical staff who cared for (him/her) while under care of hospice speak to (him/her) or you about making sure (his/her) care was consistent with (his/her) wishes? (“Yes”/“No”)  Was there any medical procedure or treatment that happened to (him/her) that was inconsistent with (his/her) previously stated wishes? (“Yes”/“No”)  Did (his/her) doctor or the medical staff who cared for (him/her) tell you about how (his/her) pain would be treated, in a way that you could understand? (“Yes”/“No”)  Was there ever a time when one doctor or nurse said one thing about treatment of (his/her) pain and another said something else? (“Yes”/“No”)  How much help in dealing with these (anxiety/sadness) feelings did [Patient] receive - less than was needed or about the right amount?  Was there ever a decision made about (his/her) care without enough input from (him/her) or (his/her) family? (“Yes”/“No”)  Was there any decision made about care or treatment that [Patient] would not have wanted?  How often was (he/she) treated with respect by those who were taking care of (him/her) – always, usually, sometimes, or never?  How often was [Patient] treated with kindness by those who were taking care of (him/her) – always, usually, sometimes, or never?  At any time while [Patient] was involved with hospice did you or your family receive any information about what to expect while (he/she) was dying? Would you have wanted (some/more) information about that? (“Yes”/“No”)  At any time while [Patient] was involved with hospice did you or your family receive any information about what to do at the time of (his/her) death? Would you have wanted (some/more) information about that? (“Yes”/“No”)  At any time while [Patient] was in the hospital did you or your family receive any information about the medicines that would be used to manage (his/her) pain, shortness of breath, or other symptoms? Would you have wanted (some/more) information about the medicines? (“Yes”/“No”)  How often were you or other family members kept informed about [Patient’s] condition – always, usually, sometimes, or never?  Was this [hospice speaking with you about your religious or spiritual beliefs] done in a sensitive manner? Did you have as much contact of that kind as you wanted? (“Yes”/“No”)  How much support in dealing with your feelings about [Patient’s] death did the doctors, nurses, and other professional staff taking care of (him/her) provide you - less support than was needed or about the right amount?  Did a doctor, nurse, or other professional staff taking care of [Patient] talk about how you might feel after [Patient’s] death? (“Yes”/“No”)   - [If yes] Was it done in a sensitive manner? - [If no] Would you have wanted them to?   Did a doctor, nurse, or other professional staff taking care of [Patient] suggest someone you could turn to for help if you were feeling stressed? (“Yes”/“No”)  Ratings from 0 (“Worst possible care”) to 10 (“Best possible care”):  How well did the doctors, nurses, and other professional staff who cared for [Patient] communicate with (him/her) and the family about the illness and the likely outcomes of care?  How would you rate how well those taking care of [Patient] provided medical care that respected (his/her) wishes?  How well did those taking care of [Patient] make sure (his/her) symptoms were controlled to a degree that was acceptable to (him/her)?  How well did those taking care of [Patient] make sure that [Patient] died with dignity - that is, died on (his/her) own terms?  How well did those taking care of [Patient] do at providing emotional support for you and [Patient’s] family and friends? | 4  1  1, 4  1, 4  1  1  1  1, 4  4  2  1, 5  1  1  3  1, 4  1, 4  1, 4  1, 4  1  1, 2  1, 4  1, 2  4  1  1  1  1, 2 |
