# Supplementary material for: Measuring the quality of patient-provider relationships in serious illness: A scoping review
Source: Palliat Med. 2025 Feb 6;39(3):332–45. doi: 10.1177/02692163251315304 (PMC11877987; doi:10.1177/02692163251315304)
Supplement: sj-docx-5-pmj-10.1177_02692163251315304 – Supplemental material for Measuring the quality of patient-provider relationships in serious illness: A scoping review [file sj-docx-5-pmj-10.1177_02692163251315304.docx]

**Denotes a measure reported by a family member, caregiver, next-of-kin, or other respondent who is not a patient/surrogate*

| **Element of relationship quality** | **Items (Measure)** |
| --- | --- |
| Engaging the patient as a whole person | Since the patient’s admission, a member of the healthcare team has asked the patient or substitute decision-maker or the patient’s family what is important to them as they consider healthcare decisions at this stage of the patient’s life (i.e., values, spiritual beliefs and other practices). (ACCEPT)  Since admission, a member of the healthcare team has asked the patient or his or her family if they had any questions or needed things clarified regarding the patient’s overall goals of care. (ACCEPT)  Since admission, a member of the healthcare team has given the patient the opportunity to express his or her fears or discuss what concerns him or her. (ACCEPT)  Since admission, a member of the healthcare team has asked the patient what treatments he or she prefers to have or not have if he or she develops a life-threatening illness. (ACCEPT)  Since admission, the patient and family have been offered support from the allied healthcare team as needed. (ACCEPT)  Providers listened to concerns. (BFS-CNH*)  Providers gave treatment patient wanted. (BFS-CNH*)  Providers were kind, caring, and respectful. (BFS-CNH*)  Providers kept family members informed about condition and treatment. (BFS-CNH*)  Providers gave enough spiritual support. (BFS-CNH*)  Over the past few days I have felt: Completely cared for…Not cared for at all. (BHI)  How satisfied are you that your doctor(s) took a personal interest in you? (CANHELP)  How satisfied are you that the doctor(s) took a personal interest in your relative? (CANHELP*)  How satisfied are you that the doctor(s) were available when you or relative needed them? (CANHELP*)  How satisfied are you that the doctors, nurses, and other healthcare professionals who looked after your relative were compassionate and supportive of you? (CANHELP*)  How satisfied are you that you were treated in a manner that preserved your sense of dignity? (CANHELP)  How satisfied are you that you were treated by those doctors, nurses, and other healthcare professionals in a manner that preserved your sense of dignity? (CANHELP*)  How satisfied are you that the care and treatment you received was consistent with your wishes? (CANHELP)  How satisfied are you that the care and treatment your relative received was consistent with his or her wishes? (CANHELP*)  How satisfied are you that the doctor(s) listened to what you had to say? (CANHELP; CANHELP*)  How satisfied are you with the way you were treated by the doctors, nurses, and other healthcare professionals looking after your relative? (CANHELP*)  How satisfied are you with your role [during the past month] in decision-making regarding your relative’s medical care? (CANHELP*)  Doctors/nurses listen to you carefully. (CES)  Doctors and nurses pay attention/Consideration was paid to relieving your concerns and worries. (CES)  Consideration was paid to relieving the patient's concerns and worries. (CES*)  Admission (use) is in accordance with the wishes of you and your family. (CES)  Admission (use) was in accordance with the wishes of the patient and family. (CES*)  Nurses helped the patient to enjoy daily life. (CES*)  The doctors gave sufficient explanation to the family about the patient's condition and the details of medical treatment. (CES*)  The doctors gave sufficient explanation to the family about the expected outcome. (CES*)  The family's wishes were respected in the selection of treatment. (CES*)  Consideration was given to the health of the family. (CES*)  Consideration was given so that the family could have their own time and continue to work. (CES*)  Was there any medical procedure or treatment that happened to (him/her) that was inconsistent with (his/her) previously stated wishes? (CEQUEL*)  Did you feel that the doctors you talked to listened to your concerns about [Patient’s] medical treatment? (CEQUEL*)  How often were you or other family members kept informed about [Patient’s] condition? (CEQUEL*)  Overall, do you feel that you and your family got as much help and support from homecare services as you needed? (CaregiverVoice Survey*)  While he/she [was being cared for by the clinician or cancer center/was receiving homecare services/was in a long-term care home/was in hospice] / During his/her last hospital admission / During the last week of life for the setting you specified, what is your assessment of the overall level of support given in the following areas: spiritual support given? respect and dignity? (CaregiverVoice Survey*)  There was enough help available to meet his/her personal care needs. (CaregiverVoice Survey*)  There was enough support to stay where he/she wanted to be. (CaregiverVoice Survey*)  Were you or his/her family given enough help and support by the healthcare providers at the actual time of his/her death? (CaregiverVoice Survey*)  Were you involved in decisions about his/her care as much as you would have wanted? (CaregiverVoice Survey*)  Were any decisions made about his/her care that he/she would not have wanted? (CaregiverVoice Survey*)  Did your healthcare providers help you, the caregiver, understand what to expect/how to prepare for his/her death? (CaregiverVoice Survey*)  My decisions about care and treatment are respected by the doctors and nurses. (CCEQ)  To what extent have your interactions with the healthcare team helped you to talk and feel understood about how cancer has affected my life? (CEQ)  How often did you see that the nurses/aides treat your family member with courtesy and respect? (CAHPS*)  Did you ever see any nurses or aides be rude to your family member or any other resident? (CAHPS*)  How often were you involved as much as you wanted to be in the decisions about your family member’s care? (CAHPS*)  Did the nurses/aides ever try to discourage you from asking questions about your family member? (CAHPS*)  How often did you get the help you needed from the hospice team during evenings, weekends, or holidays? (CAHPS*)  How often did the hospice team keep you informed about [when they would arrive to care for your family member/about your family member’s condition]? (CAHPS*)  When you or your family member asked for help from the hospice team, how often did you get help as soon as you needed it? (CAHPS*)  How often did the hospice team treat your family member with dignity and respect? (CAHPS*)  How often did the hospice team listen carefully to you when you talked with them about problems with your family member’s hospice care? (CAHPS*)  How often did the hospice team listen carefully to you? (CAHPS*)  How often do you feel your provider/doctor/nurse cares/cared about your emotional or psychological well-being? (Compassion Measure)  How often do you feel your provider/doctor/nurse is/were interested in you as a whole person? (Compassion Measure)  How often do you feel your provider/doctor/nurse is/were considerate of your personal needs? (Compassion Measure)  How often did your kidney doctors/dialysis center staff/provider listen carefully to you? (CAHPS)  How often did your kidney doctors/dialysis center staff/provider show respect for what you had to say? (CAHPS)  How often did your kidney doctors/dialysis center staff/provider really care about you as a person? (CAHPS)  How often did your kidney doctors/dialysis center staff/provider make you as comfortable as possible during dialysis? (CAHPS)  Has anyone on the dialysis center staff asked you about how your kidney disease affects other parts of your life? (CAHPS)  How was your doctor at letting you tell your story? (CARE)  How was your doctor at really listening? (CARE)  How was your doctor at being interested in you as a whole person? (CARE)  How was your doctor at fully understanding your concerns? (CARE)  Do your caregivers listen carefully to you? (CQ-index PC)  Do your caregivers take you seriously? (CQ-index PC)  Do your caregivers show interest in your personal situation? (CQ-index PC)  Do your caregivers pay attention to your relative(s)? (CQ-index PC)  Do your caregivers respect your life stance? (CQ-index PC)  Do your caregivers take your personal wishes into account? (CQ-index PC)  Describe how satisfied you were with… availability of the doctor to speak with me on a regular basis. (CCFSS*)  Describe how satisfied you were with…ability to share in the care of my family member. (CCFSS*)  Describe how satisfied you were with…support and encouragement given to me during my family member’s stay in the critical care unit. (CCFSS*)  Describe how satisfied you were with… sharing in [decisions/discussions] regarding my family member’s [care on a regular basis/recovery]. (CCFSS*)  Describe how satisfied you were with… nurses’ availability to speak with me every day about my family member’s care. (CCFSS*)  Did the clinician greet the patient *(and visitor, if present)* when entering the patient’s room? (DOC*)  Was an effort made to explain an activity to the patient *(and visitor, if present)*? (DOC*)  Was the clinician responsive to the needs or requests of the patient *(and visitor, if present)*? (DOC*)  To what extent did this clinician treat the patient with respect and dignity? (DOC*)  Your doctor/nurse respects you as an individual. (Eight-Factor Model)  You feel that your doctor/nurse will help you through the medical system to get the medical care you need. (Eight-Factor Model)  You feel that your doctor/nurse listens to what you have to say about your illness or medical treatment. (Eight-Factor Model)  [Rate] The courtesy, respect, and compassion your family member was given. (euroQ2*)  [Rate] How well the involved ICU staff made you feel that your presence was appreciated. (euroQ2*)  [Rate] How well the involved ICU staff showed an interest in your needs. (euroQ2*)  [Rate] Willingness of ICU staff to answer your questions. (euroQ2*)  [Rate] How well ICU staff provided you with explanations that you understood. (euroQ2*)  [Rate] How well ICU staff informed you about what was happening with your family member. (euroQ2*)  [Rate] How well ICU staff informed you about why things were being done to your family member. (euroQ2*)  [Rate] How well the staff involved you in major decision-making processes. (euroQ2*)  [Rate] How well ICU staff supported you when major decisions were made. (euroQ2*)  Do you think that your family member got the spiritual support he/she needed? (euroQ2*)  Was the end-of-life care according to the wishes of your family member? (euroQ2*)  Inpatient providers offered comfortable accommodations for respondents. (FATE*)  Providers listened to concerns. (FATE*)  Providers were available to talk to the patient/family. (FATE*)  Providers kept patient/family informed about the patient’s condition and treatment. (FATE*)  Patient received desired medications or treatment. (FATE*)  Patient received unwanted medication or treatment/Providers gave treatment the patient did not want. (FATE*)  Providers were kind, caring, and respectful. (FATE*)  Providers gave adequate spiritual support to patient/family. (FATE*)  Providers gave adequate emotional support to patient/family prior to death. (FATE*)  The family had enough warning one month prior to patient’s death. (FATE*)  The patient/family received enough care at home. (FATE*)  The doctor always kept you or other family members informed about resident’s condition. (FPPFC*)  Your relative’s doctor always helped you or other family members to understand what he or she was saying to you about what to expect while your relative was dying. (FPPFC*)  The doctor always spoke to you, other family members or your relative about your relative’s wishes for medical treatment at the end of life. (FPPFC*)  The doctor always listened to what you, other family members, or your relative had to say about his/her medical treatment and end-of-life care. (FPPFC*)  The doctor always understood what you, other family members, and your relative were going through. (FPPFC*)  How satisfied are you with the courtesy, respect, and compassion your family member was given? (FS-ICU*)  How satisfied are you with how well the doctors/nurses cared for your family member? (FS-ICU*)  How satisfied are you with how often doctors/nurses communicated to you about your family member’s condition? (FS-ICU*)  How satisfied are you with willingness of ICU staff to answer your questions? (FS-ICU*)  Did you feel included in the decision making process? (FS-ICU*)  Did you feel supported during the decision making process? (FS-ICU*)  Did you feel you had control over the care of your family member? (FS-ICU*)  When making decisions, did you have adequate time to have your concerns addressed and questions answered? (FS-ICU*)  How satisfied are you with the doctor’s attention to your description of symptoms? (FAMCARE)  How satisfied are you with doctor’s/palliative care team’s attention to the [patient’s/patient’s description of] symptoms? (FAMCARE*)  How satisfied are you with doctor’s attention to your description of your (care recipient’s) symptoms? (FAMCARE*)  How satisfied are you with availability of doctors/nurses/palliative care team to [your/the] family? (FAMCARE; FAMCARE*)  How satisfied are you with the way the family is included in treatment and care decisions? (FAMCARE; FAMCARE*)  How satisfied are you with the palliative care team’s response to changes in the patient’s care needs? (FAMCARE*)  How satisfied are you with emotional support provided to family members by the palliative care team? (FAMCARE*)  How satisfied are you with the way in which the patient’s physical needs for comfort are met? (FAMCARE*)  How satisfied are you with the way in which the palliative care team respects the patient’s dignity? (FAMCARE*)  I felt heard and understood by this provider and team. (Feeling Heard and Understood)  I felt this provider and team put my best interests first when making recommendations about my care. (Feeling Heard and Understood)  I felt this provider and team saw me as a person, not just someone with a medical problem. (Feeling Heard and Understood)  I felt this provider and team understood what is important to me in my life. (Feeling Heard and Understood)  How satisfied are you with the spiritual support you get from your healthcare team? (HQOL-T)  How often would you say your doctor takes the time to listen to your concerns? (THC)  To what extent does your doctor pay close attention to what you are saying? (THC)  To what extent do you think your doctor sees you as a whole person? (THC)  How much do you feel your doctor cares about you? (THC)  How often does your doctor ask how family members are coping with your illness? (THC)  How concerned do you think your doctor is about your quality of life? (THC)  Members of the care team treated [me/my loved one] with courtesy. (ICU-RESPECT)  Members of the care team made an effort to understand what matters to [me/my loved one] most. (ICU-RESPECT)  Members of the care team were attentive to [my/my loved one's] requests. (ICU-RESPECT)  [I/My loved one] felt that the care team really listened to [me/him or her]. (ICU-RESPECT)  Members of the care team made efforts to know [me/my loved one] as a unique individual. (ICU-RESPECT)  Members of the care team treated [me/my loved one] as their equal. (ICU-RESPECT)  Members of the care team treated [me/my loved one] the way they would like to be treated if they were the patient. (ICU-RESPECT)  The healthcare team’s knowledge of the patient’s wishes for end of life care. (ISC*)  [Doctor] understands my emotions, feelings, and concerns. (JSPPPE)  [Doctor] Seems concerned about me and my family. (JSPPPE)  [Doctor] can view things from my perspective. (JSPPPE)  [Doctor] asks about what is happening in my daily life. (JSPPPE)  How would you rate the friendliness and interest shown in you as a person? (KDQOL)  The care team kept my wishes at the center of my care. (LifeCourse Experience)  The care team respected me. (LifeCourse Experience)  The care team did everything they could to help with my problem or physical symptom. (LifeCourse Experience)  The care team knew my personal circumstances or situation. (LifeCourse Experience)  I had to repeat myself when telling the care team about my life. (LifeCourse Experience)  I had to repeat myself when telling the care team about my medical condition. (LifeCourse Experience)  I had to repeat myself when telling the care team about what was important to me. (LifeCourse Experience)  My goals of care include what is important to me. (LifeCourse Experience)  Before my relative left the hospital, health professionals took my preferences and those of my relative into account in deciding the place of referral to palliative care. (PCTM-C*)  Before my relative left the hospital, health professionals took my preferences and those of my relative into account in organizing the referral to palliative care. (PCTM-C*)  When my relative left the hospital, I was confident that his/her symptoms would be managed as well as possible. (PCTM-C*)  When my relative left the hospital, I was confident that we would receive all the help needed. (PCTM-C*)  Health professionals supported us throughout the referral to palliative care. (PCTM-C*)  Indicate how much of a problem or concern this has been for you within the last few days: Not feeling supported by my healthcare providers. (PDI)  Indicate how much of a problem or concern this has been for you within the last few days: Not being treated with respect or understanding by others. (PDI)  Do you feel that he/she is treating you as a human being? (PPRI)  I felt that my health concerns were understood. (PSCC-Sp)  I felt that I was treated with courtesy and respect. (PSCC-Sp)  The doctor did not take my problems very seriously. (PMH/PSQ-MD)  The doctor considered my individual needs when treating my condition. (PMH/PSQ-MD)  The doctor went straight to my medical problem without greeting me first. (PMH/PSQ-MD)  I really felt understood by my doctor. (PMH/PSQ-MD)    After my last visit with my doctor, I feel much better about my concerns. (PMH/PSQ-MD)  This doctor was interested in me as a person and not just my illness. (PMH/PSQ-MD)  It seemed to me that the doctor was not really interested in my physical well-being. (PMH/PSQ-MD)  The doctor should have shown more interest. (PMH/PSQ-MD)  Do you feel that your oncologist or cancer care providers listen to your concerns about your treatment plan? (Problem Scores)  How often do your cancer care providers allow your family/friends to ask as many questions as they want about your treatment plan? (Problem Scores)  How often do you feel that your oncologist or cancer care providers are not paying enough attention to your care? (Problem Scores)  How often do you feel that you and your family are left on your own to make sure that the right things get done for your cancer care? (Problem Scores)  How often do you feel that your cancer care providers listen to your concerns about the possible side effects of chemotherapy and/or radiation treatments? (Problem Scores)  How often do your cancer care providers do everything they can to promote your comfort during treatment? (Problem Scores)  How often are your cancer care providers available to speak with you when you have a concern about your chemotherapy? (Problem Scores)  How often is everything done to make you as comfortable as possible while you are receiving your chemotherapy treatment? (Problem Scores)  How much support do you get from the staff during chemotherapy treatments? (Problem Scores)  How often do the staff promote your dignity during chemotherapy treatments? (Problem Scores)  Overall, how often have you been treated with respect by your cancer care providers? (Problem Scores)  I have the help I wanted/needed: (QPP)   - to take care of my personal hygiene - in relation to using the toilet - to sit and lie comfortably - in relation to my meals   I have the feeling that my knowledge of my illness/disease was taken into consideration. (QPP)  I have the feeling that the doctors/nurses and assistant nurses were interested in my uplifts. (QPP)  I have the feeling that the doctors/nurses and assistant nurses were interested in my [concerns and hassles/home situation]. (QPP)  I have the feeling that the doctors/nurses and assistant nurses fully understood my situation. (QPP)  I have the feeling that I have been treated with respect by the doctors/nurses and assistant nurses. (QPP)  I have the experience that my integrity was protected when my medical records were handled. (QPP)  I have the feeling that my family and friends were treated in a positive manner by the doctors/nurses and assistant nurses. (QPP)  I have the possibility to meet with my family and friends at the times when it was most convenient for us. (QPP)  I have the feeling that my desires and needs regarding [wake-up time/bedtime/when to shower] were not restricted by the ward. (QPP)  The personnel/doctors/nurses and assistant nurses understand how I experience my situation. (QPP)  The personnel are respectful towards me. (QPP)  The personnel support me in tending to my spiritual and existential needs (life questions). (QPP)  My care is determined by my own requests and needs rather than staff procedures. (QPP)  My relatives and friends are treated with respect. (QPP)  My relatives receive the best possible help, support and care. (QPP)  My relative may participate in decisions about my care, according to my preferences. (QPP)  My relatives and friends were treated well. (QPP)  Have your carers checked how you are feeling? (Q-PAC)  Do your carers take your personal wishes into account? (Q-PAC)  Did you receive information about your relative’s condition? (Q-PAC*)  Did you get information about the pros and cons of different treatments? (Q-PAC*)  Did you get information about the impending death? (Q-PAC*)  Did the carers take each of these questions about a treatment decision seriously? (Q-PAC*)  Did you feel that the carers gave you all the help and support you needed to care for your relative? (Q-PAC*)  Did the carers ask how you were feeling? (Q-PAC*)  [Resident’s] physician knew [him/her] as a whole person. (QOD-LTC/QOD-LTC-C*)  How often have the doctors/nurses seemed distracted by other things when you talk? (QUEST)  How often have the doctors/nurses treated you more as a disease than as a person? (QUEST)  How often have the doctors/nurses showed personal concern about you? (QUEST)  How often have the doctors/nurses responded quickly in time of need? (QUEST)  How often have the doctors/nurses concern for you as an individual? (QUEST)  How good is [Doctor] at listening to what you have to say? (QOC)  How good is [Doctor] at caring about you as a person? (QOC)  How good is [Doctor] at giving [you his/her] full attention? (QOC)  How good is [Doctor] at talking [with you] about your feelings about getting sicker? (QOC)  How good is [Doctor] at asking you about important things in life? (QOC)  How good is [Doctor] at asking about spiritual or religious beliefs? (QOC)  How good is [Doctor] at including your loved ones in decisions about your illness and treatment? (QOC)  How good is [Doctor] at involving you in the decisions about the treatments that you want if you get too sick to speak for yourself? (QOC)  How good is [Doctor] at respecting the things in your life that are important to you? (QOC)  How good is [Doctor] at respecting your spiritual or religious beliefs? (QOC)  How often does the healthcare team involve you in making decisions about patient’s care? (QUAL-E*)  How often does the healthcare team keep you informed about patient’s condition? (QUAL-E*)  How often does the healthcare team respond to your concerns about [Patient]? (QUAL-E*)  Beyond my illness, my doctor has a sense of who I am as a person. (QUAL-E)  The nurses and assistant nurses provided sufficient help with daily care for the patient. (Sat-Fam-IPC*)  The patient’s and the family’s wishes were reflected in the care of the patient. (Sat-Fam-IPC*)  The patient’s dignity was protected during the care. (Sat-Fam-IPC*)  The staff was available for consultations about concerns and anxieties when death was close. (Sat-Fam-IPC*)  Complaints and requests were handled smoothly. (Sat-Fam-IPC*)  Admission was in accordance with the wishes of the patient/family. (Sat-Fam-IPC*)  The staff responded not only to physical distress but also emotional distress such as anxieties. (Sat-Fam-IPC*)  All measures were taken to keep my care recipient comfortable. (SWC-EOLD*)  The healthcare team was sensitive to my needs and feelings. (SWC-EOLD*)  I feel that all medication issues were clearly explained to me. (SWC-EOLD*)  I felt fully involved in all decision making. (SWC-EOLD*)  How successfully did your doctor/healthcare provider consider the effect of your illnesses on you and your family? (SCCCS)  How successfully did your doctor/healthcare provider listen attentively to you? (SCCCS)  How successfully did your doctor/healthcare provider treat you as a person not just as a disease? (SCCCS)  How successfully did your doctor/healthcare provider show respect for you and your family [and those important to you]? (SCCCS)  Genuine concern. (SCQ)  Saw as person. (SCQ)  Really understood needs. (SCQ)  See my perspective. (SCQ)  Did you feel that the doctors you talked to listened to your concerns about [Patient’s] medical treatment? (Toolkit After-death*)  How much information did the doctors provide you about [Patient’s] medical condition? (Toolkit After-death*)  How often did any doctor give confusing or contradictory information about [Patient’s] medical treatment? (Toolkit After-death*)  To the best of your knowledge, did [Patient’s] doctor or the medical staff who cared for (him/her) while under care of hospice speak to (him/her) or you about (his/her) wishes about medical treatment? (Toolkit After-death*)  Did (his/her) doctor or the medical staff who cared for (him/her) while under care of hospice speak to (him/her) or you about making sure (his/her) care was consistent with (his/her) wishes? (Toolkit After-death*)  Was there any medical procedure or treatment that happened to (him/her) that was inconsistent with (his/her) previously stated wishes? (Toolkit After-death*)  Did (his/her) doctor or the medical staff who cared for (him/her) tell you about how (his/her) pain would be treated, in a way that you could understand? (Toolkit After-death*)  Was there ever a decision made about (his/her) care without enough input from (him/her) or (his/her) family? (Toolkit After-death*)  Was there any decision made about care or treatment that [Patient] would not have wanted? (Toolkit After-death*)  How often was (he/she) treated with respect by those who were taking care of (him/her)? (Toolkit After-death*)  At any time while [Patient] was involved with hospice did you or your family receive any information about what to expect while (he/she) was dying? Would you have wanted (some/more) information about that? (Toolkit After-death*)  At any time while [Patient] was involved with hospice did you or your family receive any information about what to do at the time of (his/her) death? Would you have wanted (some/more) information about that? (Toolkit After-death*)  At any time while [Patient] was in the hospital did you or your family receive any information about the medicines that would be used to  manage (his/her) pain, shortness of breath, or other symptoms? Would you have wanted (some/more) information about the medicines? (Toolkit After-death*)  How often were you or other family members kept informed about [Patient’s] condition? (Toolkit After-death*)  Was this [hospice speaking with you about your religious or spiritual beliefs] done in a sensitive manner? Did you have as much contact of that kind as you wanted? (Toolkit After-death*)  How much support in dealing with your feelings about [Patient’s] death did the doctors, nurses, and other professional staff taking care of (him/her) provide you? (Toolkit After-death*)  Did a doctor, nurse, or other professional staff taking care of [Patient] talk about how you might feel after [Patient’s] death? (“Yes”/“No”) (Toolkit After-death*)   - [If yes] Was it done in a sensitive manner? - [If no] Would you have wanted them to?   Did a doctor, nurse, or other professional staff taking care of [Patient] suggest someone you could turn to for help if you were feeling stressed? (Toolkit After-death*)  How would you rate how well those taking care of [Patient] provided medical care that respected (his/her) wishes? (Toolkit After-death*)  How well did those taking care of [Patient] make sure (his/her) symptoms were controlled to a degree that was acceptable to (him/her)? (Toolkit After-death*)  How well did those taking care of [Patient] make sure that [Patient] died with dignity - that is, died on (his/her) own terms? (Toolkit After-death*)  How well did those taking care of [Patient] do at providing emotional support for you and [Patient’s] family and friends? (Toolkit After-death*) |
| Recognizing and responding to emotions | Since admission, the patient and family have been offered support from the allied healthcare team as needed. (ACCEPT)  Providers gave enough emotional support before death. (BFS-CNH*)  How satisfied are you that the emotional problems you had were adequately assessed and controlled? (CANHELP)  How satisfied are you that the emotional problems your relative had were adequately assessed and controlled? (CANHELP*)  Doctors and nurses take appropriate measures when you become depressed. (CES)  The staff took appropriate measures when the patient became depressed. (CES*)  The staff tried to give you hope/Doctors and nurses try so that your hope would be accomplished. (CES)  The staff tried so that the patient's hope could be accomplished. (CES*)  While he/she [was being cared for by the clinician or cancer center/was receiving homecare services/was in a long-term care home/was in hospice] / During his/her last hospital admission / During the last week of life for the setting you specified, what is your assessment of the overall level of support given in the following areas: emotional support? (CaregiverVoice Survey*)  When you or your family member asked for help from the hospice team, how often did you get help as soon as you needed it? (CAHPS*)  How often did your family member get the help he or she needed from the hospice team for feelings of anxiety or sadness? (CAHPS*)  How often do you feel your provider/doctor/nurse care/cared about your emotional or psychological well-being? (Compassion Measure)  How was your doctor at making you feel at ease? (CARE)  Do you receive support when you feel anxious? (CQ-index PC)  Do you receive support when you feel depressed? (CQ-index PC)  Describe how satisfied you were with…support and encouragement given to me during my family member’s stay in the critical care unit. (CCFSS*)  Describe how satisfied you were with… sensitivity of the doctor(s) to my family member’s needs. (CCFSS*)  Your primary doctor tells you bad news in a sensitive and caring manner. (Eight-Factor Model)  [Rate] How well the involved ICU staff provided emotional support. (euroQ2*)  Do you think that your family member got the emotional support he/she needed? (euroQ2*)  Providers gave adequate emotional support to patient/family prior to death. (FATE*)  How satisfied are you with emotional support provided to the patient by the palliative care team? (FAMCARE*)  How satisfied are you with emotional support provided to family members by the palliative care team? (FAMCARE*)  How satisfied are you with the emotional support you get from your healthcare team? (HQOL-T)  How often does your doctor offer hope? (THC)  [Doctor] understands my emotions, feelings, and concerns. (JSPPPE)  Is the relationship with your doctor important for your emotional well-being? (PPRI)  It seemed to me that the doctor was not really interested in my emotional well-being. (PMH/PSQ-MD)  Was your prognosis presented to you in a sensitive manner? (Problem Scores)  How much emotional support did you receive from your oncologist or cancer care providers when your prognosis was discussed with you? (Problem Scores)  I have the feeling that the doctors/nurses and assistant nurses were interested in my uplifts. (QPP)  I have the feeling that the doctors/nurses and assistant nurses showed sympathy when I was suffering. (QPP)  I receive the best possible help for [depression/anxiety]. (QPP)  How often have the doctors/nurses ignored your feelings? (QUEST)  How good is [Doctor] at talking [with you] about your feelings about getting sicker? (QOC)  Attention was continuously paid to the patient’s suffering. (Sat-Fam-IPC*)  The staff responded not only to physical distress but also emotional distress such as anxieties. (Sat-Fam-IPC*)  The healthcare team was sensitive to my needs and feelings. (SWC-EOLD*)  How successfully did your doctor/healthcare provider express sensitivity, caring and compassion for your situation? (SCCCS)  How successfully did your doctor/healthcare provider strive to understand your emotional needs? (SCCCS)  Communicated sensitive. (SCQ)  Provided comfort. (SCQ)  How much help in dealing with these (anxiety/sadness) feelings did [Patient] receive? (Toolkit After-Death*)  How much support in dealing with your feelings about [Patient’s] death did the doctors, nurses, and other professional staff taking care of (him/her) provide you? (Toolkit After-Death*)  Did a doctor, nurse, or other professional staff taking care of [Patient] suggest someone you could turn to for help if you were feeling stressed? (Toolkit After-Death*)  How well did those taking care of [Patient] do at providing emotional support for you and [Patient’s] family and friends? (Toolkit After-Death*) |
| Fostering therapeutic alliance | Since admission, a member of the healthcare team has asked the patient or his or her family if they had any questions or needed things clarified regarding the patient’s overall goals of care. (ACCEPT)  Providers were kind, caring, and respectful. (BFS-CNH*)  Over the past few days I have felt: Completely cared for…Not cared for at all. (BHI)  How satisfied are you that your doctor(s) were available when you needed them? (CANHELP)  How satisfied are you that the doctor(s) were available when you or relative needed them? (CANHELP*)  How satisfied are you with the level of trust and confidence you had in the doctor(s)/nurses who looked after you? (CANHELP)  How satisfied are you with the level of trust and confidence you had in the doctor(s)/nurses who looked after your relative? (CANHELP*)  How satisfied are you that the doctors and nurses were compassionate and supportive? (CANHELP)  How satisfied are you that the doctors, nurses, and other healthcare professionals who looked after your relative were compassionate and supportive of him or her? (CANHELP*)  How satisfied are you that the doctor(s) listened to what you had to say? (CANHELP)  Doctors/nurses listen to you carefully. (CES)  Doctors/nurses understand you adequately. (CES)  Doctors/nurses develop a good relationship with you. (CES)  The staff at the hospital are friendly and make me feel at ease. (CCEQ)  At times I have felt abandoned by the medical staff. (CCEQ)  How often did you see that the nurses/aides treat your family member with kindness? (CAHPS*)  How often did you feel that the nurses/aides/hospice team really cared about your family member? (CAHPS*)  Did you ever see any nurses or aides be rude to your family member or any other resident? (CAHPS*)  How often do you feel your provider/doctor/nurse is/were able to gain your trust? (Compassion Measure)  How often did your kidney doctors/dialysis center staff/provider listen carefully to you? (CAHPS)  How often did your kidney doctors/dialysis center staff/provider spend enough time with you? (CAHPS)  How often do you feel your [provider/doctor/nurse] shows/showed / How was the doctor at showing / How did the physician primarily responsible for your treatment show you care and compassion? (Compassion Measure; CARE)  Did you feel comfortable asking the dialysis center staff everything you wanted about dialysis care? (CAHPS)  How often did dialysis center staff behave in a professional manner? (CAHPS)  Were you ever unhappy with the care you received at the dialysis center or from your kidney doctors? [If yes] How often were you satisfied with the way they handled these problems? (CAHPS)  What number would you use to rate the kidney doctors you have now? (CAHPS)  What number would you use to rate your dialysis center staff? (CAHPS)  How often did dialysis center staff check you as closely as you wanted while you were on the dialysis machine? (CAHPS)  How was your doctor at letting you tell your story? (CARE)  How was your doctor at really listening? (CARE)  How was your doctor at being positive? (CARE)  Are your caregivers polite to you? (CQ-index PC)  Do your caregivers listen carefully to you? (CQ-index PC)  Do your caregivers have enough time for you? (CQ-index PC)  Do your caregivers take you seriously? (CQ-index PC)  Do your caregivers have a ‘warm’ attitude? (CQ-index PC)  Do you have the opportunity to talk to your caregivers about how you are feeling? (CQ-index PC)  Do you receive help in good time when you are in need of care? (CQ-index PC)  Are you offered help in good time in acute situations? (CQ-index PC)  Did the clinician greet the patient *(and visitor, if present)* when entering the patient’s room? (DOC*)  Did the clinician make an attempt to be at the patient’s eye level when not engaged in activities that require standing? (DOC*)  How would you describe the general demeanor of the clinician? (DOC*)  Would you describe the clinician’s demeanor as pleasant? (DOC*)  Would you describe the clinician’s demeanor as compassionate? (DOC*)  Would you describe the clinician’s demeanor as supportive? (DOC*)  Would you describe the clinician’s demeanor as distant? (DOC*)  You feel that your doctor/nurse will help you through the medical system to get the medical care you need. (Eight-Factor Model)  You feel that your doctor/nurse listens to what you have to say about your illness or medical treatment. (Eight-Factor Model)  You have complete trust in your doctor/nurse. (Eight-Factor Model)  [Rate] The courtesy, respect, and compassion your family member was given. (euroQ2*)  Providers were kind, caring, and respectful. (FATE*)  Inpatient providers handled the patient gently. (FATE*)  Inpatient providers supervised the patient closely enough. (FATE*)  The doctor always listened to what you, other family members, or your relative had to say about his/her medical treatment and end-of-life care. (FPPFC*)  How satisfied are you with the courtesy, respect, and compassion your family member was given? (FS-ICU*)  How satisfied are you with availability of doctor to the patient/your (care recipient)? (FAMCARE*)  How satisfied are you with the availability of nurses/doctors to answer your questions? (FAMCARE)  I felt this provider and team put my best interests first when making recommendations about my care. (Feeling Heard and Understood)  How often would you say your doctor takes the time to listen to your concerns? (THC)  To what extent does your doctor pay close attention to what you are saying? (THC)  How much do you like your doctor? (THC)  How much do you trust your doctor? (THC)  How thorough is your doctor? (THC)  How much do you respect your doctor? (THC)  How much do you feel your doctor cares about you? (THC)  How often does your doctor ask how you are coping with cancer? (THC)  Members of the care team treated [me/my loved one] with courtesy. (ICU-RESPECT)  [I/My loved one] felt that the care team really listened to [me/him or her]. (ICU-RESPECT)  Members of the care team treated [me/my loved one] as their equal. (ICU-RESPECT)  Members of the care team treated [me/my loved one] the way they would like to be treated if they were the patient. (ICU-RESPECT)  [Doctor] is an understanding doctor. (JSPPPE)  [Doctor] devotes enough time to me. (JSPPPE)  I trusted my care team. (LifeCourse Experience)  The care team spent the right amount of time with me. (LifeCourse Experience)  I was able to get in touch with someone on my care team when needed. (LifeCourse Experience)  Indicate how much of a problem or concern this has been for you within the last few days: Not feeling supported by my healthcare providers. (PDI)  Do you feel comfortable when you ask him/her questions and require explanations? (PPRI)  Do you think that the nursing personnel is efficient and helpful? (PPRI)  Do you feel that everything possible is being done for you? (PPRI)  I felt encouraged to talk about my personal health concerns. (PSCC-Sp)  I felt I had enough time with my doctor. (PSCC-Sp)  The doctor went straight to my medical problem without greeting me first. (PMH/PSQ-MD)  I feel the doctor did not spend enough time with me. (PMH/PSQ-MD)  The doctor was not friendly to me. (PMH/PSQ-MD)  I would not recommend this doctor to a friend. (PMH/PSQ-MD)  The doctor seemed to brush off my questions. (PMH/PSQ-MD)  Were you allowed to ask as many questions as you wanted about the prognosis of your cancer? (Problem Scores)  How often are your cancer care providers available to speak with you when you have a concern about your chemotherapy? (Problem Scores)  How much support do you get from the staff during chemotherapy treatments? (Problem Scores)  Have you a good relationship with your healthcare providers? (QCQ-EOL)  I have the feeling that the doctors/nurses and assistant nurses exhibited a sense of commitment. (QPP)  I have the feeling that the doctors/nurses and assistant nurses were personal in their contact with me. (QPP)  I have a feeling of confidence that I received sincere answers to my questions from the doctors/nurses and assistant nurses. (QPP)  I have the feeling that I was treated in a positive manner by the doctors/nurses and assistant nurses. (QPP)  The doctors/nurses and assistant nurses showed commitment; ‘cared about me’. (QPP)  There was a nurse or aide with whom [Resident] felt comfortable. (QOD-LTC/QOD-LTC-C*)  [Resident] had a physician whom [he/she] trusted. (QOD-LTC/QOD-LTC-C*)  How often have the doctors/nurses spent enough time with you? (QUEST)  How often have the doctors/nurses seemed distracted by other things when you talk? (QUEST)  How often have the doctors/nurses been willing to take time to listen? (QUEST)  How satisfied have you been with your doctors’/nurses’ bedside manner? (QUEST)  How satisfied have you been with your doctors’/nurses’ way of talking to you? (QUEST)  How good is [Doctor] at looking you in [the] eye? (QOC)  How good is [Doctor] at listening to what you have to say? (QOC)  How good is [Doctor] at giving you [his/her] full attention? (QOC)  Overall, how would you rate this doctor’s communication with you? (QOC)  The ward staff provided enough time in the care of the patient. (Sat-Fam-IPC*)  The demeanor of the ward staff was always warm. (Sat-Fam-IPC*)  How successfully did your doctor/healthcare provider listen attentively to you? (SCCCS)  How successfully did your doctor/healthcare provider gain your trust? (SCCCS)  How successfully did your doctor/healthcare provider spend enough time with you? (SCCCS)  Feel cared for. (SCQ)  Attentive. (SCQ)  Very supportive. (SCQ)  Provided care. (SCQ)  Spoke with kindness. (SCQ)  Behaved in caring way. (SCQ)  Good relationship. (SCQ)  Warm presence. (SCQ)  Sincere. (SCQ)  How often was [Patient] treated with kindness by those who were taking care of (him/her)? (Toolkit After-Death*) |
| Promoting information exchange | Since admission, a member of the healthcare team has asked the patient or his or her family if they had any questions or needed things clarified regarding the patient’s overall goals of care. (ACCEPT)  Providers kept family members informed about condition and treatment. (BFS-CNH*)  How satisfied are you that the doctor(s) explained things relating to your illness in a straight-forward, honest manner? (CANHELP)  How satisfied are you that the doctor(s) explained things relating to your relative’s illness in a straight-forward, honest manner? (CANHELP*)  How satisfied are you that the doctor(s) explained things related to your illness in a way you could understand? (CANHELP)  How satisfied are you that the doctor(s) explained things relating to your relative’s illness in a way you could understand? (CANHELP*)  How satisfied are you that you received consistent information about your condition from all doctors/nurses [looking after you]? (CANHELP)  How satisfied are you that you received consistent information about your relative’s condition from all the doctors and nurses looking after him or her? (CANHELP*)  How satisfied are you that you received updates about your condition, treatments, test results, etc. in a timely manner? (CANHELP)  How satisfied are you that you received updates about your relative’s condition, treatments, test results, etc. in a timely manner? (CANHELP*)  How satisfied are you with discussions with your doctor(s) about where you would be cared for if [you were to get worse/your condition worsened]? (CANHELP)  How satisfied are you with discussions with the doctor(s) about where your relative would be cared for if he or she were to get worse? (CANHELP*)  How satisfied are you with discussions with your doctor(s) about the use of life sustaining technologies? (CANHELP)  How satisfied are you with discussions with the doctor(s) about the use of life sustaining technologies? (CANHELP*)  The doctors gave sufficient explanations to you about your present condition and the details of your medical treatment. (CES)  The doctors gave sufficient explanation to the patient about their present condition and the details of medical treatment. (CES*)  The doctors gave sufficient explanation to the family about the patient's condition and the details of medical treatment. (CES*)  The doctors give sufficient explanations to you about the expected outcome. (CES)  The doctors gave sufficient explanation to the patient about the expected outcome. (CES*)  The doctors gave sufficient explanation to the family about the expected outcome. (CES*)  Was there ever a problem understanding what any doctor was saying to you about what to expect from treatment? (CEQUEL*)  How often were you or other family members kept informed about [Patient’s] condition? (CEQUEL*)  Did your healthcare providers help you, the caregiver, understand what to expect/how to prepare for his/her death? (CaregiverVoice Survey*)  I feel there is enough time to ask questions when I come to clinic. (CCEQ)  The doctors are very open and will tell you anything you need to know. (CCEQ)  I am content with the information I have received about my diagnosis. (CCEQ)  I am content with the information I have received about my prognosis. (CCEQ)  If I have questions about new treatments, my doctor is happy to discuss these with me. (CCEQ)  How often did you get this information [about your family member from a nurse/aide] as soon as you wanted? (CAHPS*)  How often did the nurses/aides/hospice team explain things in a way that was easy for you to understand? (CAHPS*)  Did the nurses/aides ever try to discourage you from asking questions about your family member? (CAHPS*)  How often did the hospice team keep you informed about [when they would arrive to care for your family member/your family member’s condition]? (CAHPS*)  How often did anyone from the hospice team give you confusing or contradictory information about your family member’s condition or care? (CAHPS*)  Did the hospice team give you as much information as you wanted about what to expect while your family member was dying? (CAHPS*)  How often was the information you were given about your family member by the nursing home staff different from the information you were given by the hospice team? (CAHPS*)  How often did your kidney doctors/dialysis center staff/provider explain things in a way that was easy to understand? (CAHPS)  Did either your kidney doctors or dialysis center staff talk to you as much as you wanted about which treatment is right for you? (CAHPS)  How was your doctor at explaining things clearly? (CARE)  Do your caregivers explain things to you in a way you could understand? (CQ-index PC)  Do your caregivers give you contradictory information? (CQ-index PC)  Describe how satisfied you were with… honesty of the staff about my family member’s condition. (CCFSS*)  Describe how satisfied you were with… availability of the doctor to speak with me on a regular basis. (CCFSS*)  Describe how satisfied you were with…clear explanation of tests, procedures, and treatments. (CCFSS*)  Describe how satisfied you were with… clear answers to my questions. (CCFSS*)  Describe how satisfied you were with… nurses’ availability to speak with me every day about my family member’s care. (CCFSS*)  Was an effort made to explain an activity to the patient *(and visitor, if present)*? (DOC*)  Your doctor/nurse has given you clear information about what to expect regarding your illness and outlook. (Eight-Factor Model)  Your doctor/nurse has given you clear information about the risks and side effects of your treatment. (Eight-Factor Model)  Your doctor/nurse has given you a clear explanation of your treatment alternatives. (Eight-Factor Model)  [Rate] Willingness of ICU staff to answer your questions. (euroQ2*)  [Rate] How well ICU staff provided you with explanations that you understood. (euroQ2*)  [Rate] Perceived honesty of information provided to you about your family member’s condition. (euroQ2*)  [Rate] How well ICU staff informed you about what was happening with your family member. (euroQ2*)  [Rate] How well ICU staff informed you about why things were being done to your family member. (euroQ2*)  [Rate] The consistency of information provided to you about your family member’s condition. (euroQ2*)  [Rate] The overall quality of information provided to you by [doctors/nurses]. (euroQ2*)  Providers gave contradictory information. (FATE*)  Providers spoke in an understandable way. (FATE*)  Providers kept patient/family informed about the patient’s condition and treatment. (FATE*)  The family had enough warning one month prior to patient’s death. (FATE*)  The doctor always kept you or other family members informed about resident’s condition. (FPPFC*)  Your relative’s doctor always helped you or other family members to understand what he or she was saying to you about what to expect while your relative was dying. (FPPFC*)  How satisfied are you with how often doctors/nurses communicated to you about your family member’s condition? (FS-ICU*)  How satisfied are you with willingness of ICU staff to answer your questions? (FS-ICU*)  How satisfied are you with the honesty of information provided to you about your family member’s condition? (FS-ICU*)  How well ICU staff informed you what was happening to your family member and why things were being done? (FS-ICU*)  How satisfied are you with the consistency of information provided to you about your family member’s condition? (FS-ICU*)  When making decisions, did you have adequate time to have your concerns addressed and questions answered? (FS-ICU*)  How satisfied are you with information provided about [prognosis/side effects (of treatment)/the patient’s/your care recipient’s tests/how to manage the patient’s/your care recipient’s pain/symptoms]? (FAMCARE*)  How satisfied are you with family conferences held to discuss the patient’s illness/meetings with the palliative care team to discuss the patient’s condition and plan of care? (FAMCARE*)  How satisfied are you with the way in which the patient’s condition and likely progress have been explained by the palliative care team? (FAMCARE*)  How satisfied are you with doctor’s/palliative care team’s attention to [the] patient’s symptoms/description of symptoms? (FAMCARE*)  How satisfied are you with doctor’s attention to your description of your (care recipient’s) symptoms? (FAMCARE*)  How satisfied are you with the doctor’s attention to your description of symptoms? (FAMCARE)  How satisfied are you with information given about your prognosis/tests/side effects/how to manage pain? (FAMCARE)  How satisfied are you with the availability of nurses/doctors to answer your questions? (FAMCARE)  How much of the time would you say your doctor is honest with you? (THC)  To what extent do you feel comfortable asking your doctor questions? (THC)  How often do you understand your doctor’s explanations and suggestions? (THC)  The quality of the discussions with the healthcare team about the use of life-sustaining technologies (ISC*)  The care team helped me understand all of my options when I had a choice about my care. (LifeCourse Experience)  I received easy to understand information from the care team in response to my questions. (LifeCourse Experience)  I had unanswered questions about how my illness affected my everyday life. (LifeCourse Experience)  I had unanswered questions about how my illness affected my health. (LifeCourse Experience)  Do you feel that your doctor is informing you clearly and sincerely about your disease and about the therapy you are undergoing? (PPRI)  My questions were answered to my satisfaction. (PSCC-Sp)  I was able to get the advice I needed about my health issues. (PSCC-Sp)  The doctors seemed to communicate well about my care. (PSCC-Sp)  The doctor did not give me all the information I thought I should have been given. (PMH/PSQ-MD)  The doctor used words I did not understand. (PMH/PSQ-MD)  I understand my illness much better after seeing this doctor. (PMH/PSQ-MD)  The doctor should have told me more about how to care for my condition. (PMH/PSQ-MD)  The doctor told me to call back if I had any questions or problems. (PMH/PSQ-MD)  I felt the doctor was being honest with me. (PMH/PSQ-MD)  Was your prognosis explained to you in a way that you could understand? (Problem Scores)  Were you allowed to ask as many questions as you wanted about the prognosis of your cancer? (Problem Scores)  How often have you been given confusing or contradictory information about your prognosis from your oncologist or other cancer care providers? (Problem Scores)  How often does your oncologist or cancer care provider explain your test results in a way that you can understand? (Problem Scores)  How often do you want more information about your test results than you receive from your cancer care providers? (Problem Scores)  How often have you been given confusing or contradictory information about your test results from your cancer care providers and/or other physicians involved in your care? (Problem Scores)  How often do your cancer care providers allow your family/friends to ask as many questions as they want about your treatment plan? (Problem Scores)  How often do your cancer care providers explain what they are doing to do in a way that you can understand? (Problem Scores)  Have healthcare providers given you clear explanations about your health status? (QCQ-EOL)  I have satisfactory information regarding: (QPP)   - [Medical examinations and tests/medical treatments], so that I understood their relevance as well as how they would be implemented - The drugs I needed, so that I understood their effects, and how they should be administered - Routine issues of the ward (e.g. visiting hours, meals, rounds, etc.) - The [medical examination and test results/medical treatment results] - Self-care procedures   I have a feeling of confidence that I received sincere answers to my questions from the doctors/nurses and assistant nurses. (QPP)  The personnel [seem to] give me honest answers to my questions. (QPP)  I receive(d) useful information on: (QPP)   - How examinations and treatments would take place - The results of examinations and treatments - How care and treatments will take place - The effects and use of medicine - My illness and my symptoms - What I may expect in the near future (development of the illness and symptoms, my health and function) - How to take care of myself/self-care; ‘how I should take care of myself’ Which doctor are responsible for my medical care - Which nurses are responsible for my nursing care   Are you getting enough information about…? (Q-PAC)   - Diagnosis - The course of the disease - With regard to end-of-life care   Do your carers explain things to you understandably? (Q-PAC)  Do your carers give you conflicting information? (Q-PAC)  Did you receive information about your relative’s condition? (Q-PAC*)  Did you get information about the pros and cons of different treatments? (Q-PAC*)  Did you get information about the impending death? (Q-PAC*)  Did the carers take each of these questions about a treatment decision seriously? (Q-PAC*)  [Resident] had as much information as [he/she] wanted about [his/her] illness. (QOD-LTC/QOD-LTC-C*)  How good is [Doctor] at using words you understand? (QOC)  How good is [Doctor] at answering all questions about your illness [and treatment]? (QOC)  How good is [Doctor] at talking [to you] about details if you got sicker? (QOC)  How good is [Doctor] at talking [to you] about how long you have to live? (QOC)  How good is [Doctor] at talking [to you] about what dying might be like? (QOC)  How comfortable do you feel your doctor is [in] talking about dying? (QOC)  Overall, how would you rate this doctor’s communication with you? (QOC)  How often does the healthcare team keep you informed about patient’s condition? (QUAL-E*)  The physician explained the treatment to alleviate the patient’s discomfort. (Sat-Fam-IPC*)  The physician’s explanations were easy to understand. (Sat-Fam-IPC*)  I feel that all medication issues were clearly explained to me. (SWC-EOLD*)  How successfully did your doctor/healthcare provider convey information in a way that is understandable? (SCCCS)  How successfully did your doctor/healthcare provider communicate test results in a timely and sensitive manner? (SCCCS)  Was there ever a problem understanding what any doctor was saying to you about what to expect from treatment? (Toolkit After-Death*)  How much information did the doctors provide you about [Patient’s] medical condition? (Toolkit After-Death*)  How often did any doctor give confusing or contradictory information about [Patient’s] medical treatment? (Toolkit After-Death*)  Did (his/her) doctor or the medical staff who cared for (him/her) tell you about how (his/her) pain would be treated, in a way that you could understand? (Toolkit After-Death*)  Was there ever a time when one doctor or nurse said one thing about treatment of (his/her) pain and another said something else? (Toolkit After-Death*)  At any time while [Patient] was involved with hospice did you or your family receive any information about what to expect while (he/she) was dying? Would you have wanted (some/more) information about that? (Toolkit After-Death*)  At any time while [Patient] was involved with hospice did you or your family receive any information about what to do at the time of (his/her) death? Would you have wanted (some/more) information about that? (Toolkit After-Death*)  At any time while [Patient] was in the hospital did you or your family receive any information about the medicines that would be used to manage (his/her) pain, shortness of breath, or other symptoms? Would you have wanted (some/more) information about the medicines? (Toolkit After-Death*)  How often were you or other family members kept informed about [Patient’s] condition? (Toolkit After-Death*)  Did a doctor, nurse, or other professional staff taking care of [Patient] talk about how you might feel after [Patient’s] death? (Toolkit After-Death*)   - [If yes] Was it done in a sensitive manner - [If no] Would you have wanted them to?   How well did the doctors, nurses, and other professional staff who cared for [Patient] communicate with (him/her) and the family about the illness and the likely outcomes of care? (Toolkit After-Death*) |
| Sharing decision-making | How satisfied are you with your role in decision making regarding your medical care? (CANHELP)  How satisfied are you with your role [during the past month] in decision-making regarding your relative’s medical care? (CANHELP*)  Consideration is given so that you can participate in the selection of treatment. (CES)  Consideration was given so that the patient could participate in the selection of treatment. (CES*)  Was he/she involved in decisions about his/her care as much as he/she would have wanted? (CaregiverVoice Survey*)  Were you involved in decisions about his/her care as much as you would have wanted? (CaregiverVoice Survey*)  Was he/she given the opportunity to discuss advance care planning with his/her healthcare providers? (CaregiverVoice Survey*)  I am given the opportunity to discuss my treatment plan with the doctors. (CCEQ)  To what extent have your interactions with the healthcare team helped you to freely discuss my concerns about cancer and my treatment options? (CEQ)  How often were you involved as much as you wanted to be in the decisions about your family member’s care? (CAHPS*)  Did either your kidney doctors or dialysis center staff talk to you as much as you wanted about which treatment is right for you? (CAHPS)  Were you as involved as much as you wanted in choosing the treatment for kidney disease that is right for you? (CAHPS)  How was your doctor at making a plan of action with you? (CARE)  Do your caregivers give you the chance to plan your own day? (CQ-index PC)  Are you involved in decisions about your care? (CQ-index PC)  Describe how satisfied you were with…ability to share in the care of my family member. (CCFSS*)  Describe how satisfied you were with… sharing in [decisions/discussions] regarding my family member’s [care on a regular basis/recovery]. (CCFSS*)  You feel that you are able to participate in decisions about your care. (Eight-Factor Model)  [Rate] How well the staff involved you in major decision-making processes. (euroQ2*)  Did you feel included in the decision making process? (FS-ICU*)  How satisfied are you with the way the family is included in treatment and care decisions? (FAMCARE; FAMCARE*)  The care team helped me make a choice about my care when I had one. (LifeCourse Experience)  My goals of care include what is important to me. (LifeCourse Experience)  I felt included in decisions about my health. (PSCC-Sp)  I have the possibility to participate in the decision-making process regarding my medical/personal care. (QPP)  I have good opportunity to participate in the decisions that apply/applied to [my care/medical care/nursing care/my individual plan for my care/choosing where to receive my care]. (QPP)  My relative may participate in decisions about my care, according to my preferences (QPP)  Can you co-decide about your care? (Q-PAC)  [Resident] participated as much as [he/she] wanted in the decisions about [his/her] care. (QOD-LTC/QOD-LTC-C*)  How good is [Doctor] at involving you in treatment discussions about your care? (QOC)  How good is [Doctor] at including your loved ones in decisions about your illness and treatment? (QOC)  How good is [Doctor] at involving you in the decisions about the treatments that you want if you get too sick to speak for yourself? (QOC)  How often does the healthcare team involve you in making decisions about patient’s care? (QUAL-E*)  I participate as much as I want in the decisions about my care. (QUAL-E)  I felt fully involved in all decision making. (SWC-EOLD*)  How successfully did your doctor/healthcare provider always involve you in decisions about your treatment? (SCCCS)  Was there ever a decision made about (his/her) care without enough input from (him/her) or (his/her) family? (Toolkit After-Death*) |
| Enabling self-management and patient navigation | To what extent have your interactions with the healthcare team helped you deal with changes in my relationships as a result of cancer? (CEQ)  To what extent have your interactions with the healthcare team helped you explore better ways to communicate with my healthcare team, my family and others? (CEQ)  To what extent have your interactions with the healthcare team helped you clarify your values and beliefs? (CEQ)  To what extent have your interactions with the healthcare team helped you talk about my concerns about the future and to be less frightened? (CEQ)  To what extent have your interactions with the healthcare team helped you better express and manage feelings? (CEQ)  How was your doctor at helping you take control? (CARE)  Do your caregivers give you the chance to plan your own day? (CQ-index PC)  Dialysis staff encourage me to be as independent as possible. (KDQOL)  Dialysis staff support me in coping with my kidney disease. (KDQOL)  The care team helped me understand what was important to me. (LifeCourse Experience)  The personnel support [and assist] me in living [the rest of] my life in a meaningful way. (QPP)  My care is/was determined by my own requests and needs rather than staff procedures. (QPP)  Did your carers give you freedom to plan your day? (Q-PAC)  Although [he/she] could not control certain aspects of [his/her] illness, [Resident] had a sense of control about [his/her] treatment decisions. (QOD-LTC/QOD-LTC-C*)  Although I cannot control certain aspects of my illness, I have a sense of control about my treatment decisions. (QUAL-E) |
